# Liquid-metal-based three-dimensional microelectrode arrays integrated with implantable ultrathin retinal prosthesis for vision restoration

**DOI:** 10.1038/s41565-023-01587-w

**Published:** 2024-01-15

**Authors:** Won Gi Chung, Jiuk Jang, Gang Cui, Sanghoon Lee, Han Jeong, Haisu Kang, Hunkyu Seo, Sumin Kim, Enji Kim, Junwon Lee, Seung Geol Lee, Suk Ho Byeon, Jang-Ung Park

**Affiliations:** 1 Department of Materials Science & Engineering, Yonsei University, Seoul, Republic of Korea; 2 Center for Nanomedicine, Institute for Basic Science (IBS), Seoul, Republic of Korea; 3 Graduate Program of Nano Biomedical Engineering (NanoBME), Advanced Science Institute, Yonsei University, Seoul, Republic of Korea; 4 Institute of Vision Research, Department of Ophthalmology, Severance Eye Hospital, Yonsei University College of Medicine, Seoul, Republic of Korea; 5 Brain Korea 21 Project for Medical Science, Yonsei University College of Medicine, Seoul, Republic of Korea; 6 School of Chemical Engineering, Pusan National University, Busan, Republic of Korea; 7 Institute of Vision Research, Department of Ophthalmology, Gangnam Severance Hospital, Yonsei University College of Medicine, Seoul, Republic of Korea; 8 Department of Organic Material Science and Engineering, Pusan National University, Busan, Republic of Korea; 9 Department of Neurosurgery, Yonsei University College of Medicine, Seoul, Republic of Korea

**Keywords:** Biomaterials, Electronic devices, Biomaterials

## Abstract

Electronic retinal prostheses for stimulating retinal neurons are promising for vision restoration. However, the rigid electrodes of conventional retinal implants can inflict damage on the soft retina tissue. They also have limited selectivity due to their poor proximity to target cells in the degenerative retina. Here we present a soft artificial retina (thickness, 10 μm) where flexible ultrathin photosensitive transistors are integrated with three-dimensional stimulation electrodes of eutectic gallium–indium alloy. Platinum nanoclusters locally coated only on the tip of these three-dimensional liquid-metal electrodes show advantages in reducing the impedance of the stimulation electrodes. These microelectrodes can enhance the proximity to the target retinal ganglion cells and provide effective charge injections (72.84 mC cm^−2^) to elicit neural responses in the retina. Their low Young’s modulus (234 kPa), owing to their liquid form, can minimize damage to the retina. Furthermore, we used an unsupervised machine learning approach to effectively identify the evoked spikes to grade neural activities within the retinal ganglion cells. Results from in vivo experiments on a retinal degeneration mouse model reveal that the spatiotemporal distribution of neural responses on their retina can be mapped under selective localized illumination areas of light, suggesting the restoration of their vision.

## Abstract (web summary)

A soft artificial retina with flexible phototransistors and three-dimensional liquid-metal microelectrodes is used to enhance proximity to retinal ganglion cells and minimize damage to soft tissue as well as improve charge injection for vision restoration in retinal degenerative in vivo models.

## Main

Retinal degenerative diseases, including retinitis pigmentosa and age-related macular degeneration, can cause gradual loss or permanent damage to photoreceptor cells, resulting in severe vision impairment^1,2^. However, the inner retinal neurons (ganglion and bipolar cells) can be preserved despite photoreceptor degeneration.

An electronic retinal prosthesis, which electrically stimulates inner retinal neurons using photoresponsive devices, has emerged as a promising method to restore vision^3–6^. The electrical activation of retinal neurons can generate visual perceptions (phosphene)^7–13^. This device has been adapted to human subjects blind by retinal degeneration, although still being limited by low visual acuity. The subretinal prosthesis, placed between the retinal pigment epithelium and the degenerated photoreceptor layer, provides stable mechanical fixation of the device, but has a greater degree of surgical difficulty with a limited implant size. The risks associated with subretinal implantation also include residual photoreceptor loss and retinal pigment epithelium disruption. Although subretinal implantation is routinely done in vitreoretinal surgery, the epiretinal prosthesis, placed inside the vitreous and facing the retinal ganglion cell (RGC) side, has shown promise in both long- and short-term clinical observations. However, the results have proven that one of the main limitations is caused by the unconformities between the retina and the implant, since the threshold to elicit retinal responses depends on the electrode–cell distance^13–18^. Low proximity resulting from these unconformities can also induce the lateral spread of the electric field, decreasing the spatial resolution of stimulation^19,20^. This imprecise stimulation on the epiretinal surface can excite the RGC axons, which traverse between the device and RGCs, generating irregular visual perceptions to patients^21^. To enhance stimulation resolution and minimize axonal stimulation, it is important to establish a precise and stable contact and reduce the distance between the target RGC bodies and the stimulation electrodes, thereby reducing the activation thresholds of the RGC somas. However, patients with severe retinal degenerative diseases have locally non-uniform retinal surfaces, which can create an undesired geometrical gap between the retinal surface and stimulation electrodes^19,22^.

To address this limitation, ultrathin and flexible optoelectronics have been studied to conformally attach them to the curved retinal surface^23^. Despite the substantial flexibility of these devices, the flat-shaped electrodes cause a geometrical gap from the locally bumpy retina surface. In this regard, three-dimensional (3D) microelectrodes show promise for effectively stimulating the nervous system, reducing this electrode–cell distance. Also, they can stimulate selective local areas by bypassing neurons that should not be stimulated, providing excellent selectivity and high spatial resolution^24^. However, previous 3D neural electrodes were formed using rigid solid-state materials, with substantial mechanical mismatch at their interface with soft biological tissues. This can directly damage the soft retina, or cause inflammatory responses within the retina^23,25–27^.

Here we introduce a soft artificial retina where flexible, ultrathin and photosensitive transistor arrays are integrated with the soft 3D stimulation electrodes of liquid metals (LMs) for vision restoration. First, compared with subretinal implantation, this soft artificial retina is implanted using a safer epiretinal implantation method with less invasiveness to the retina. Second, soft and biocompatible LMs were 3D printed as stimulation electrodes with high resolutions. Studies of LMs including their properties (low modulus and infinite elastic limit) and processing methods (printing and patterning) have been conducted to utilize them in electronics^28–31^. However, research on LM as a biointerfacing material is in the early stages. Gallium-based LMs, such as eutectic gallium–indium alloy (EGaIn), are intrinsically soft and exhibit low toxicity (Supplementary Fig. 1)^32–36^. Compared with previous pillar/spike electrodes using rigid materials, these soft 3D stimulation electrodes exhibit low moduli, minimizing the undesired damage to the retina^26,37–39^. Also, platinum (Pt) nanoclusters locally coated on the tip of these LM electrodes show advantages with regard to effectively injecting charges into the retinal neurons. Third, machine learning is applied to the output signals produced during the animal experiments, so the evoked RGC spikes can be analysed. Last, the in vivo experiments confirmed that the signal amplification due to visible-light illumination induces real-time responses in the RGCs of the local area where the light is incident for live retinal degenerative (rd1) mice with massive photoreceptor degeneration, suggesting the restoration of their vision.

## Soft artificial retina with 3D LM microelectrode arrays

**Fig. 1:**
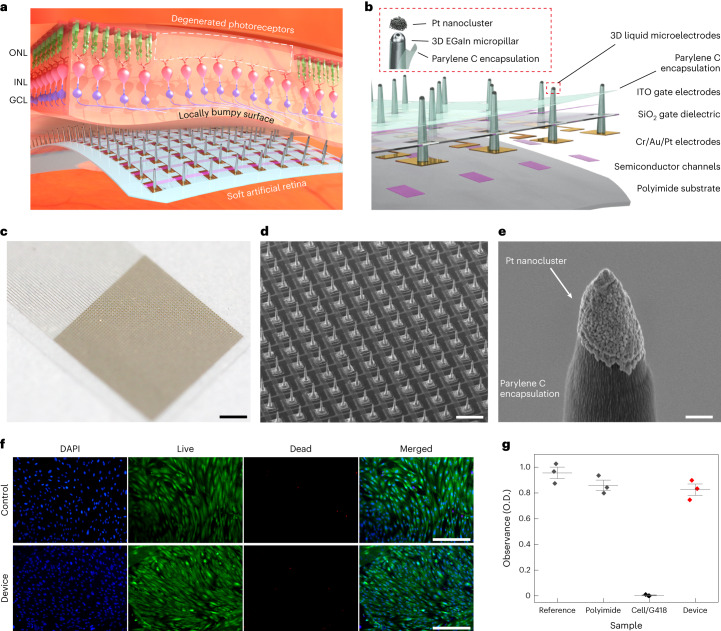
Soft artificial retina with 3D LM microelectrode arrays. **a**, Schematic of the artificial retina integrated with the 3D LM microelectrodes in close proximity to the locally bumpy, retinal surface, due to the degeneration of photoreceptors. ONL, INL and GCL indicate the outer nuclear layer, inner nuclear layer and ganglion cell layer, respectively. **b**, Schematic of the layouts of the artificial retina based on the integration of photosensitive transistors with 3D LM microelectrodes. **c**, Photograph of the artificial retina where a high-resolution transistor array was integrated with the 3D LM microelectrodes. Scale bar, 1 mm. **d**, Scanning electron microscopy (SEM) image of the high-resolution phototransistor arrays (50 × 50 pixels; pixel pitch, 100 μm) with 3D LM microelectrodes of 60 μm height before depositing the top parylene C encapsulating layer. Scale bar, 100 μm. This experiment was repeated five times with similar results. **e**, SEM image of the Pt nanoclusters (PtB) locally coated only on the tip of the 3D LM stimulation electrode. Scale bar, 1 μm. This experiment was independently repeated more than ten times with similar results. **f**, Representative fluorescence microscopy images of DAPI and live/dead staining of human retina cells cultured on the device with 3D LM microelectrodes. Scale bars, 400 μm. This experiment was independently repeated three times with similar results. **g**, Cell viability of the artificial retina. O.D., optical density. Data are mean ± s.d. with *n* = 3 independent experiments. Source data

**Extended Data Fig. 1:**
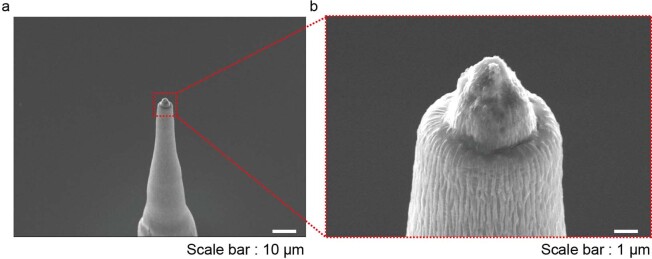
SEM images of the 3D-printed liquid-metal pillar with their tip selectively opened. **a**, SEM image of the 3D-printed liquid-metal pillar with their tip selectively opened using anisotropic O_2_ RIE before the electroplating of platinum nanoclusters. Scale bar, 10 mm. **b**, magnified SEM view of this tip. Scale bar, 1 mm. This experiment was repeated more than 10 times independently with similar results. Source data

Figure 1a shows an artificial retina with 3D LM microelectrodes in close proximity to the non-uniform, degenerative retinal surface. This ultrathin and flexible device was conformably laminated on the innermost retinal surface, and the protrudent pillar-like probes of soft LMs directly stimulated the RGCs. Figure 1b illustrates the schematic of the device layout. The 3D EGaIn micropillar array was directly printed on the drain electrode surfaces of the phototransistors to form the stimulation electrodes at room temperature. Then, the pillars’ sidewalls were encapsulated by the parylene C layer. The pillars’ tips were opened using anisotropic O_2_ reactive ion etching (RIE) as the charge injection sites to the retinal tissue before the electroplating of Pt nanoclusters, denoted as Pt black (PtB) (Extended Data Fig. 1). Methods describes the detailed fabrication processes. This PtB coating adds nanometre-scale roughness to these 3D LM microelectrodes, substantially increasing their electrochemical surface area. When external light is illuminated onto the phototransistor, a photocurrent is generated within the semiconductor channel, resulting in the amplification of its drain current (*I*_D_). This amplified *I*_D_ generated due to the incident light suggests the substantially increased charge injection into RGCs through the 3D LM microelectrode with pulsed stimuli of drain voltage (*V*_D_). Then, the action potentials evoked within the RGCs can be delivered to the optic nerve, thereby substituting the visual information. Figure 1c shows this artificial retina where a high-resolution transistor array was integrated with the 3D LM microelectrodes. In this sample, these 3D LM microelectrodes (height, 60 μm; diameter, 20 μm) were formed in every drain electrode of this transistor array (Fig. 1d and Supplementary Fig. 2). Figure 1e shows the locally coated PtB on the 3D LM microelectrode’s tip. This negligibly changed the elastic modulus of the 3D printed EGaIn, which was comparable with biological tissues and significantly lower than the rigid, solid-state electrodes (Supplementary Fig. 3). On the other hand, the long-term implantation of these conventional electrodes has been limited due to the considerable damage to the delicate retina tissue when interfacing them. Although the Argus implant was shown to function ten years post-implantation, discrepancies between the retina surface and the device’s rigid components have been reported. Such mismatches increased the geometrical gap between them, leading to a substantial decrease in impedance but an increase in stimulation threshold, limiting the effective retinal stimulation^40^.

We conducted a cell viability test using live/dead cell assay with human retinal pigment epithelium cells. The differences in the portion of live/dead cells between the device and reference for seven days were negligible (Fig. 1f). The percentage of cell survival of the device was 82% (Fig. 1g). We also conducted an in vitro apoptosis assay using flow cytometry and 98.3% of the cells cultured on the device were live cells (Supplementary Fig. 4). These results satisfy the in vitro cytotoxicity standard (>80%) for medical devices^41^.

To ensure the in vivo biocompatibility of the device with 3D LM microelectrodes, various tests, regarding immune and neurotoxicity, were conducted five weeks post-implantation in live rd1 mice (*n* = 3). First, five-week monitoring of the fundus showed no signs of bleeding, inflammation or cataracts despite the device implantation (Supplementary Fig. 5). Next, an immunohistochemical assay revealed no decline in fluorescence indicative of RGC and no accumulation of macrophages and microglia (Supplementary Fig. 6). The 3D rendered images taken by the confocal imaging of the whole-mount mouse retina on the device also showed no accumulation of macrophages and microglia around them (Supplementary Fig. 7). Third, the device was implanted onto the retinal surface without tilting or collapsing with 3D LM microelectrode tips positioned on the RGC layer with neither malignancy nor inflammation (Supplementary Fig. 8a). Last, we confirmed that the device did not significantly influence the retinal thickness (Supplementary Fig. 8b). Supplementary Methods provides more details regarding the biocompatibility of the artificial retina.

## Characterizations of the soft artificial retina

**Fig. 2:**
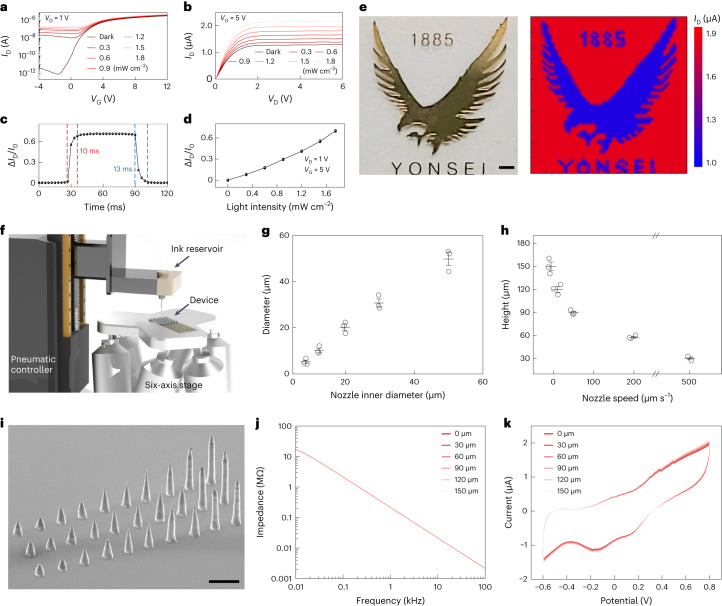
Optoelectronic properties of the phototransistor arrays and electrochemical properties of the 3D LM microelectrode. **a**, Transfer characteristics under stepwise irradiation of light (*V*_D_ = 1 V). **b**, Output characteristics under stepwise irradiation of light (*V*_G_ = 5 V). **c**, Response time of the phototransistor arrays at *V*_D_ = 1 V and *V*_G_ = 5 V under the irradiation of light (470 nm, intensity of 1.8 mW cm^−2^). **d**, Relative changes in *I*_D_ of the phototransistor arrays as a function of light intensities (*V*_D_ = 1 V, *V*_G_ = 5 V, *I*_0_ is *I*_D_ in the dark state and Δ*I*_D_ = *I*_D_ – *I*_0_). The slope of the plot represents the light responsivity of the device. **e**, Photograph of the eagle-shaped shadow mask (left) and contour plot (right) of *I*_D_ under the irradiation of light (1.8 mW cm^−2^). Scale bar, 500 μm. **f**, Schematic of the high-resolution direct printing system. **g**, Diameter of the 3D LM microelectrodes as a function of the inner diameters of the nozzles. Data are mean ± s.d. with *n* = 3 independent printings. **h**, Height of the 3D LM microelectrodes as a function of the speed of the six-axis stage (inner nozzle diameter, 20 μm). Data are mean ± s.d. with *n* = 3 independent printings. **i**, SEM image of the printed EGaIn pillar array with varying heights before the deposition of PtB. Scale bar, 100 μm. This experiment was independently repeated more than ten times with similar results. **j**, Impedance spectroscopy of 3D LM microelectrodes with various heights. **k**, Cyclic voltammogram of 3D LM microelectrodes with various heights. Source data

The current–voltage (*I*–*V*) characteristics (Fig. 2a,b) of the phototransistor in the artificial retina showed typical light-sensitive field-effect transistor behaviours with different light intensities (Supplementary Fig. 9). The field-effect mobility at ambient conditions was calculated as ∼341 cm^2^ V^−1^ s^−1^. The on/off ratio (*I*_on_/*I*_off_) and threshold voltage (*V*_th_) were 1.61 × 10^6^ and 2.6 V, respectively (Supplementary Fig. 10). The phototransistor exhibited a rapid response and recovery time of 10 and 13 ms, respectively, with negligible hysteresis (Fig. 2c). The relative change in *I*_D_ of this phototransistor (Δ*I*_D_/*I*_0_) was linearly proportional to the incident-light intensities (Fig. 2d; *I*_0_ is *I*_D_ at the dark state and Δ*I*_D_ = *I*_D_ *–* *I*_0_). Supplementary Fig. 11 provides the relative change in *I*_D_ as a function of light wavelength. The phototransistor array allowed the visualization of light passing through an eagle-shaped shadow mask pattern during light illumination (Fig. 2e and Supplementary Methods).

**Extended Data Fig. 2:**
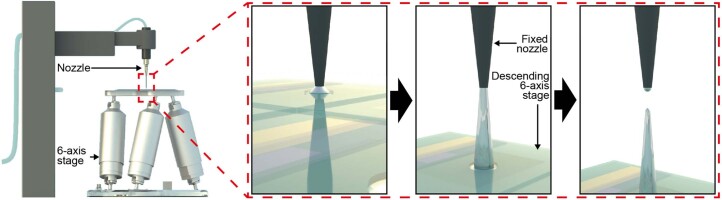
The 3D-printing process of the liquid-metal pillar. Schematic illustrations of the 3D-printing process to form a liquid-metal pillar.

To fabricate a pillar-shaped 3D LM microelectrode array as the stimulation electrodes, we used a direct printing method with a high-resolution printing system (Fig. 2f). Methods describes the detailed printing processes. The EGaIn pillar’s diameter could be determined by the inner diameter of the glass capillary nozzle (Fig. 2g). The pillar height could be formed in a controllable manner by adjusting the vertical descending speed of the stage (Fig. 2h,i and Extended Data Fig. 2). Also, the printed pillars, using different nozzle diameters, exhibited sufficient heights to target the retinal neurons (Supplementary Fig. 12).

**Extended Data Fig. 3:**
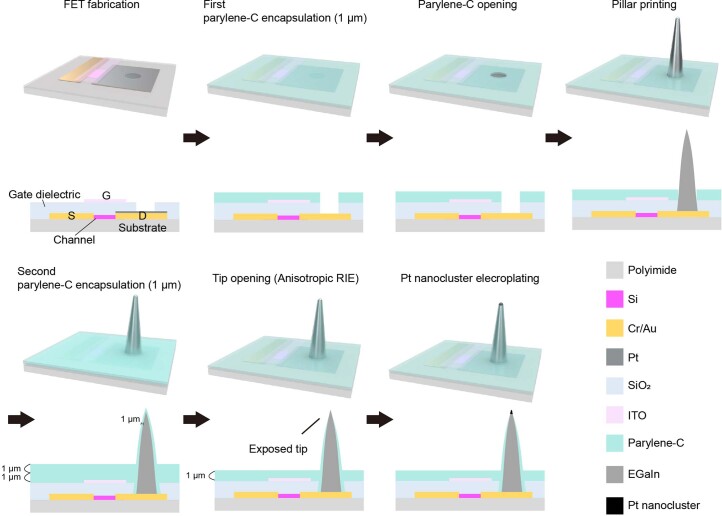
Detailed fabrication process of the 3D microelectrode. Schematic illustrations of the 3D microelectrode fabrication process.

PtB was locally coated only onto their EGaIn tips using an electroplating method (Extended Data Fig. 3 and Methods) and the 3D LM microelectrodes were reliably attached to the drain electrodes during post-printing processes (Supplementary Fig. 13). Electrochemical impedance spectroscopy and cyclic voltammetry analysis were conducted to compare the impedance and charge storage capacity characteristics of these electrodes (Supplementary Fig. 14). The dimension of their PtB-coated tips was identical (diameter, 4 μm; geometrical surface area, 25.12 μm^2^), with only difference in their heights. These 3D PtB-coated EGaIn pillar (PtB/EGaIn) electrodes exhibited an impedance of ∼210 kΩ (at 1 kHz), approximately three times lower than that of the PtB-uncoated case (Supplementary Fig. 15). The impedance and charge storage capacity (∼72.84 mC cm^−2^), calculated from the cyclic voltammetry curves, did not notably vary with the pillar height (Fig. 2j,k). We compared the impedance (at 1 kHz) and the charge storage capacity with materials used for neural interfaces (Supplementary Figs. 16 and 17). These height-independent properties can be advantageous for the consistent stimulation of target cells using 3D LM microelectrodes with varying heights. We recorded the stimulating pulse using the recording electrode, placed adjacent to the stimulation electrode, and confirmed that the pulse amplitude increased proportionally with the light intensity, validating the device as a light-responsive stimulator (Supplementary Fig. 18).

## Ex vivo electrophysiological experiments

**Fig. 3:**
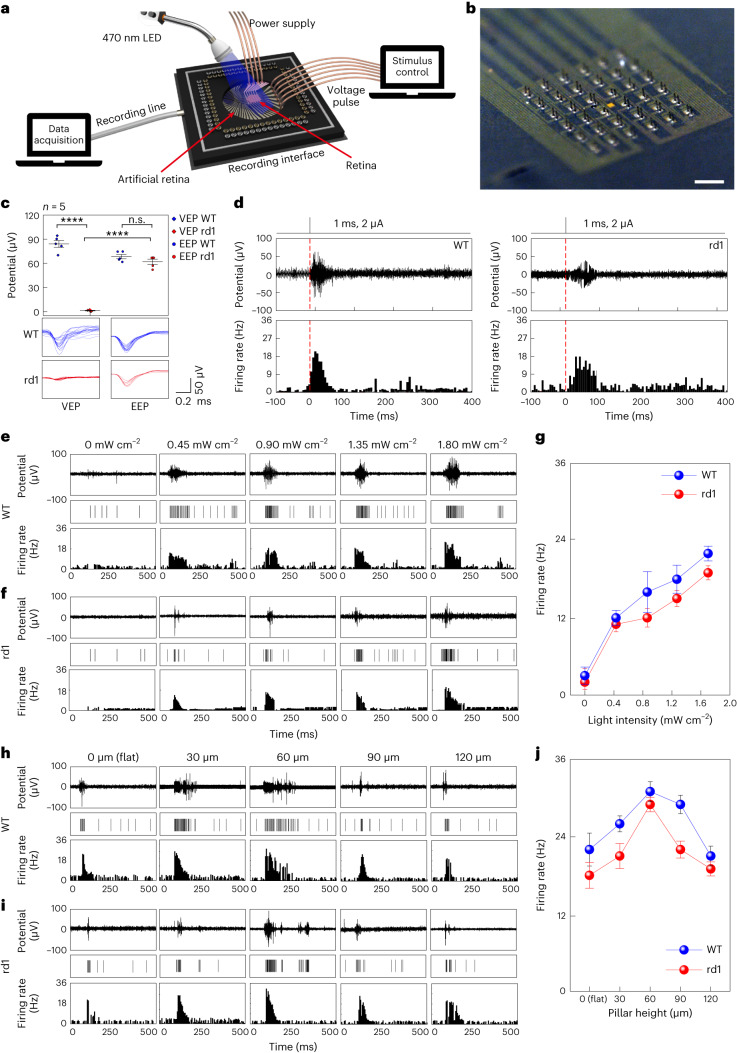
Ex vivo experiment using WT and rd1 mouse retina. **a**, Schematic of the experimental setup for an ex vivo experiment using WT and rd1 mouse retina. **b**, Optical stereomicrograph of a device consisting of 36 stimulating and recording 3D LM microelectrode pairs (pitch, 40 μm). Scale bar, 400 μm. **c**, VEP and EEP of WT and rd1 mouse retina. Data are mean ± s.d. with *n* = 5 biologically independent mice. Significance was calculated using an unpaired one-tailed *t*-test: *P* = 2.860441 (n.s.), *P* = 0.0000234 (****, left), *P* = 0.000017 (****, right). **d**, EEPs of WT and rd1 mouse retina (pulse width, 1 ms; current, 2 μA) in the dark state. The red dashed line indicates the initiation of stimulation. **e**, EEPs of WT mouse retina under light (470 nm) exposure with different intensities during the operation of the artificial retina with flat-surface-type stimulation electrodes. **f**, EEPs of rd1 mouse retina under light (470 nm) exposure with different intensities during the operation of the artificial retina with flat-surface-type stimulation electrodes. **g**, Firing rates of EEPs as a function of illuminated light intensities for WT and rd1 mouse retina. Data are mean ± s.d. with *n* = 5 biologically independent mice. **h**, EEPs of WT mouse retina under light (470 nm) exposure during the operation of artificial retina with different heights of the 3D LM microelectrode. **i**, EEPs of rd1 mouse retina under light (470 nm) exposure during the operation of the artificial retina with different heights of the 3D LM microelectrode. **j**, Firing rates of evoked RGC spikes as a function of heights of 3D LM microelectrodes for WT and rd1 mouse retina. Data are mean ± s.d. with *n* = 5 biologically independent mice. Source data

The responses in both wild-type (WT) and rd1 mice retinas (*n* = 5) were tested by electrical stimulation using the artificial retina with 3D LM microelectrodes (height, 60 μm; Fig. 3a). For recording the visually or electrically evoked retinal responses, each recording electrode was positioned adjacent (pitch, 40 μm) to each stimulation electrode (Fig. 3b). The isolated retinas from WT and rd1 mice were placed on the device, with 3D LM microelectrodes directed towards the RGC side of the retina. Methods provides the experimental details of this ex vivo experiment. Before implantation, the device was instantly frozen to turn the liquid-phase EGaIn into a solid by leaving it in cold storage (below the melting point of EGaIn, that is, ∼15.7 °C). The 3D LM microelectrodes returned to the liquid phase and did not collapse post-implantation into the retina. For electrical stimulation, the transistor of the device was operated with a specific condition (*V*_G_, d.c. bias of 5 V; *V*_D_, pulsed bias of 1 V with a duration of 1 ms and frequency of 10 Hz), and the recordings were performed with the adjacent recording electrodes. Since mice are dichromatic mammals with only two cone types (blue and green light sensitive)^42,43^, blue light (470 nm) was used. The visually evoked potentials (VEPs) were recorded under this light exposure without device operation, whereas electrically evoked potentials (EEPs) were recorded with device operation in the dark state. The light did not induce retinal responses within the rd1 mouse retina (Fig. 3c). However, electrical stimulation during device operation could elicit RGC spikes with a comparable EEP magnitude in both WT (68 μV) and rd1 (62 μV) mouse retinas. When a single-pulse electrical stimulation was delivered to both WT and rd1 mice retina (in the dark state), the recorded retinal activities of rd1 mice showed an earlier and more pronounced increase in firing activity compared with that of the WT case (Fig. 3d). The morphological changes in the rd1 mice retina, including a reduction in RGC size and thickness of the inner nuclear layer, are known to affect the functional properties of RGCs, resulting in an increased stimulation threshold and prolonged latency^44^. The EEPs were elicited during device operation using a flat-surface-type electrode (height, 0 μm) for WT and rd1 mice retinas, respectively, during light illumination with different intensities (Fig. 3e,f). The firing rates of the evoked RGC spikes increased proportionally to the light intensity in both WT and rd1 mice retinas (Fig. 3g). The WT mice retina showed a higher firing rate owing to the native responses from its normal photoreceptor layer, compared with the rd1 case.

For comparison with the flat-surface-type electrode case, similar experiments were also conducted using a device with various heights of 3D LM microelectrodes (Fig. 3h,i). Despite the height-independent electrochemical properties, the pillar-structured electrodes increased the firing activities of RGCs during electrical stimulation (Fig. 3j). The firing rate decreased again as the pillar’s height exceeded 90 μm. Considering the retinal layers’ thickness (Supplementary Table 1), this could be caused by the mistargeting of RGCs as the stimulating tip passed through the target RGCs. Although changing the polarity may stimulate the mistargeted cells, fabricating 3D LM microelectrodes with optimal heights allows the electrode tips to directly interface with the target cells.

## Signal processing using machine learning

**Fig. 4:**
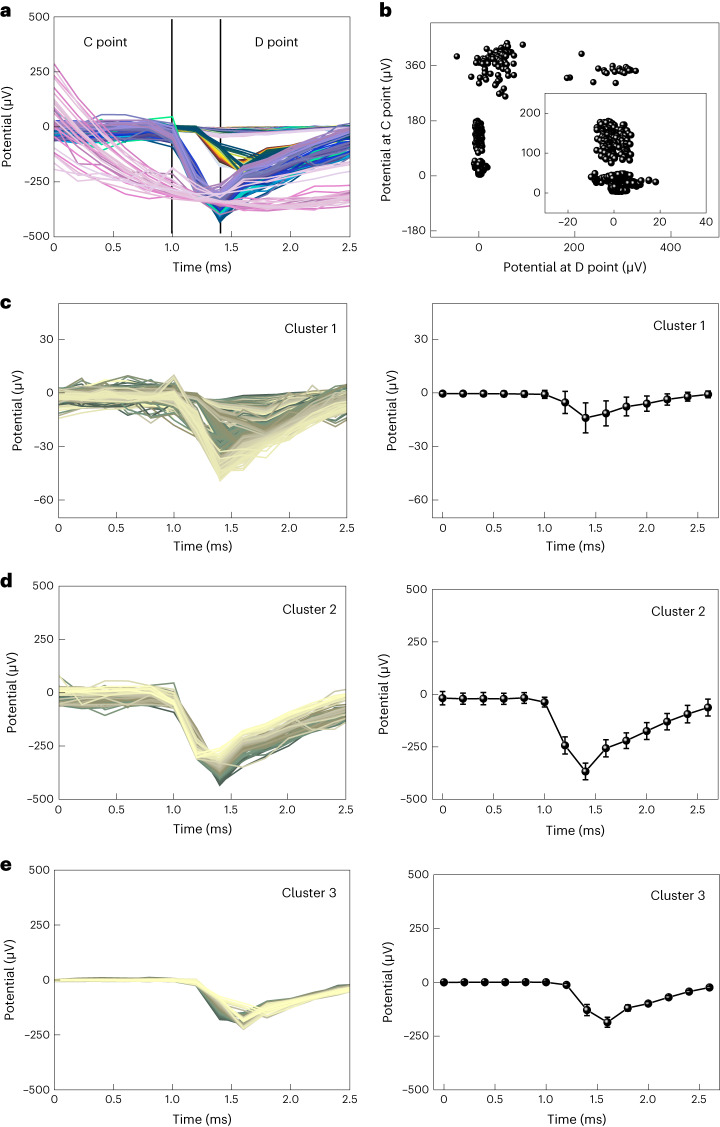
Signal classification using unsupervised machine learning. **a**, Primarily classified evoked RGC spikes from hierarchical clustering. The black lines represent the points C and D, which provide the criteria for secondary clustering. **b**, Classified datasets from *K*-means clustering. **c**, Entire data of evoked RGC spikes classified as cluster 1, sorted on the basis of their magnitude and shape (left), as well as their mean RGC spikes of cluster 1 (right). Data are mean ± s.d. with *n* = 5 biologically independent mice. **d**, Entire data of the evoked RGC spikes classified as cluster 2, sorted on the basis of their magnitude and shape (left), and their mean RGC spikes of cluster 2 (right). Data are mean ± s.d. with *n* = 5 biologically independent mice. **e**, Entire data of the evoked RGC spikes classified as cluster 3, sorted on the basis of their magnitude and shape (left), as well as their mean RGC spikes of cluster 3 (right). Data are mean ± s.d. with *n* = 5 biologically independent mice.

Considering the complexity of the retinal activities, we utilized unsupervised machine learning for this signal processing^45–48^. For the primary categorization, 4,992 spikes were classified through hierarchical clustering, by setting the retinal spike values as the input data. The given data were classified into distinctive clusters according to the magnitude and shape of the signal (Fig. 4a). Then, we conducted *K*-means clustering^49^, with primarily categorized signals as the input data. The number of clusters (*K*) was optimized to 4 through the elbow method and silhouette coefficient, where the points C and D marked in the graph were used for the criteria of classification^48,49^. As a result, the retinal spike data were further classified into four clusters with different magnitudes of potential values (Fig. 4b and Supplementary Methods). These classified retinal spikes were analysed by unsupervised machine learning to obtain the average signals with their standard deviations. The signals within the same cluster for clusters 1, 2 and 3 showed a similar form and temporal duration of potential values (Fig. 4c–e).

When stimulating the RGC soma, a typical extracellularly recorded spiking response shows a rapid decrease (depolarization) followed by an increase (repolarization) in the membrane potentials, whereas RGC axons show the opposite spiking response^50^. The waveforms of the sorted RGC signals, recorded right after electrical stimulation with 3D LM microelectrodes, only present the somatic RGC responses with sub-millisecond depolarization. These results indicate the potential for the selective stimulation of RGC somas using 3D LM microelectrodes. Although axonal stimulation cannot be eliminated, this selective stimulation of the RGC somas holds the potential to reduce axonal activation, thereby leading to a more natural vision with less irregular perception.

## In vivo vision restoration of live rd1 mice

**Fig. 5:**
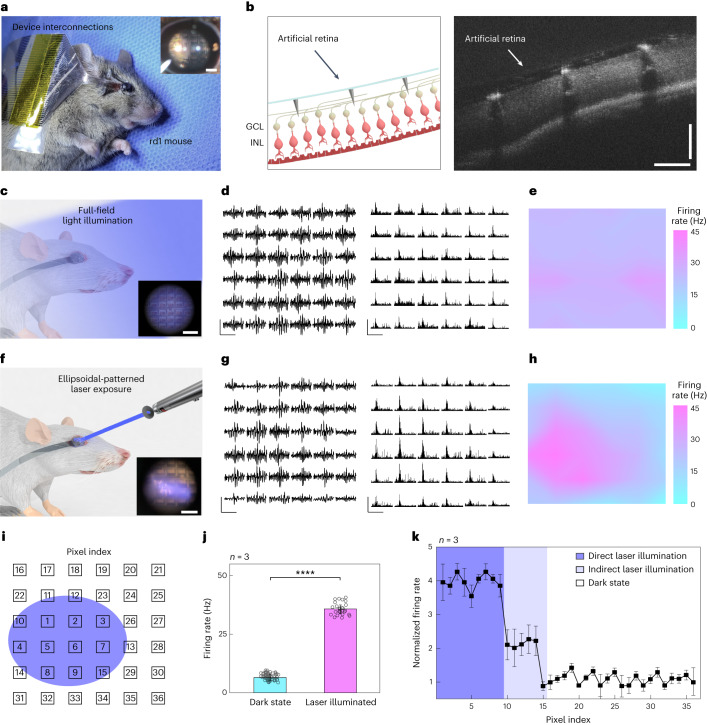
In vivo experiment using live rd1 mice for vision restoration. **a**, Photograph of the in vivo experimental setup using the live rd1 mouse. The inset shows the fundus image of a live rd1 mouse with device implantation. Scale bar, 1 mm. **b**, Schematic (left) and optical coherence tomography image (right) of the retina of the rd1 mouse after device implantation. Scale bars, 100 μm. **c**, Schematic of the in vivo experiment under full-field blue-light illumination (wavelength, 470 nm; intensity, 1.80 mW cm^−2^). The inset shows the fundus image of a live rd1 mouse with the implanted device under full-field blue-light illumination. The distorted image is due to light refraction from the artificial vitreous body. Scale bar, 200 μm. **d**, Spike train and firing rate of the evoked RGC spikes during device operation under constant full-field blue-light illumination. Scale bars, 200 ms (horizontal); 100 μV (left, vertical); 40 Hz (right, vertical). **e**, Contour plot of firing rates of the evoked RGC spikes under full-field blue-light illumination. **f**, Schematic of the in vivo animal experiment under continuous laser exposure (wavelength, 415 nm; intensity, 1.80 mW cm^−2^) through an ellipsoidal-patterned shadow mask. The inset shows the fundus image of a live rd1 mouse with the implanted device under continuous laser exposure through an ellipsoidal-patterned shadow mask. Scale bar, 200 μm. **g**, Spike train and firing rate of the evoked RGC spikes during device operation under continuous laser exposure through an ellipsoidal-patterned shadow mask. Scale bars, 200 ms (horizontal); 100 μV (left, vertical); 40 Hz (right, vertical). **h**, Contour plot of firing rates of the evoked RGC spikes under the illumination of patterned laser. **i**, Index of pixels of the artificial retina. The coloured ellipsoid indicates the local illumination spot of a laser. **j**, Firing rates of all the pixels for the laser-illuminated and laser-unilluminated state (*n* = 3). The error bars denote s.d. Significance was calculated using an unpaired one-tailed *t*-test: *P* < 0.0001 (****). **k**, Normalized firing rate with three different laser illumination states in every pixel. Data are mean ± s.d. with *n* = 3 biologically independent mice. Source data

An artificial retina device with 3D LM microelectrodes was implanted into the live rd1 mice in vivo (*n* = 3). Before this experiment, we confirmed that the photoreceptor layer was fully degenerated (Supplementary Fig. 19). After attaching the device with external device interconnections (Supplementary Fig. 20), either (1) full-field illumination (470 nm) or (2) a continuous laser exposure (415 nm) through an ellipsoidal-patterned shadow mask was applied to the eye fundus. This device was well attached to the retinal surface with no notable damage or bleeding (Fig. 5a). The cross-sectional optical coherence tomography image obtained after this surgery indicates that the 3D LM microelectrodes were conformally surrounded by retinal tissues without their collapse (Fig. 5b). We further investigated the collapse of 3D LM microelectrodes post-implantation, by measuring the pillars’ tilted angle from optical coherence tomography images after implanting 180 electrodes in total into the rd1 mice retina (*n* = 5), and validated that the implanted electrodes did not collapse (Supplementary Fig. 21).

**Extended Data Fig. 4:**
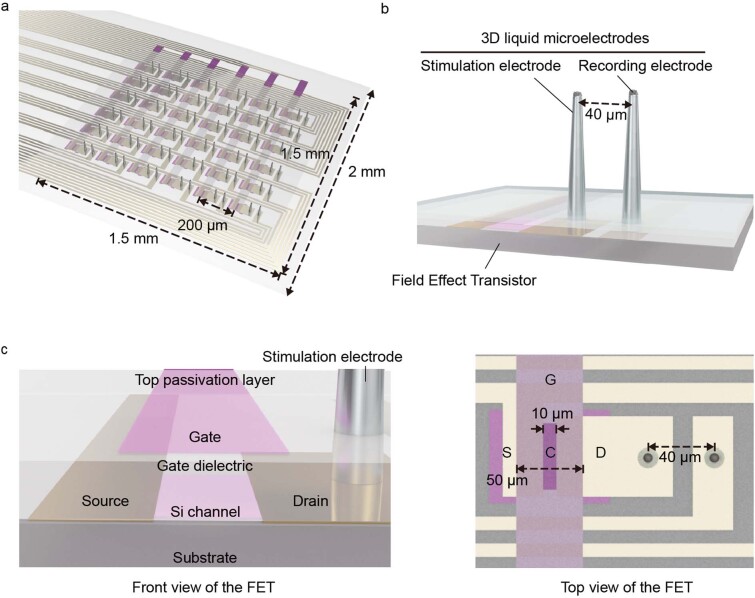
Schematic illustrations of design of the soft artificial retina device. Schematic illustrations of **a**, the design of our artificial retina device, **b**, the pixel design, **c**, the layout and dimensions of a FET.

The EEPs were recorded by projecting visible light onto its retina after implanting the artificial retina (Fig. 5c and Extended Data Fig. 4). Real-time traces of potentials and firing rates of the evoked spikes under constant light illumination were recorded, and the firing rates were spatially mapped during this illumination (Fig. 5d,e). The recorded responses showed consistent potential magnitudes and firing rates of evoked spikes compared with the case involving no light illumination (Supplementary Figs. 22 and 23), indicating good uniformity across the stimulated retinal area using the device.

One of the key challenges to develop artificial vision restoration is object recognition. We demonstrated the responses from the degenerative retina, by selectively exposing the localized areas using a laser through an ellipsoidal-patterned shadow mask (Fig. 5f). The light-illuminated local area exhibited relatively larger retinal responses, compared with the area in the dark state (Fig. 5g). When the RGC axons are electrically stimulated, the antidromic propagation of electrical stimuli can occur, leading to the misguided RGC response in the dark state. However, the spatial distribution on the maximum firing rates (that is, the receptive field) was very similar to the ellipsoidal shape of this illumination (Fig. 5h). Also, the sorted RGC spikes (Supplementary Fig. 24) show a typical waveform of the somatic RGC response similar to the ex vivo results (Fig. 4). To quantitatively compare the retinal responses in the laser-illuminated and laser-unilluminated area (that is, the dark state), the position of each recording electrode (pixel) was marked as an index (Fig. 5i). Then, the maximum firing rates recorded from the fully illuminated pixels (indexes 1–9) and the dark-state pixels (indexes 16–36) were averaged. The RGC activity in the fully exposed areas was approximately fourfold higher than the background RGC activity.

## Conclusions

We have reported a soft artificial retina consisting of high-resolution, flexible phototransistor arrays with directly printed 3D LM microelectrodes capable of minimally invasive retinal stimulation. In vivo experiments demonstrated that the visible-light illumination induced spiking activities in the RGCs of the local retina area where the light was incident, suggesting the potential for vision restoration in live rd1 mice. These results can have a prognostic meaning in the development of personalized artificial retinas for patients with uneven retinal degeneration.

**Extended Data Fig. 5:**
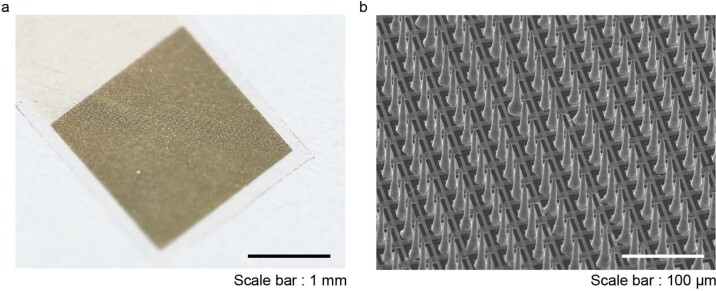
High-resolution artificial retina device. **a**, Photograph of the artificial retina where a high-resolution transistor array was integrated with 3D liquid-metal electrodes (250 × 250 pixels). Scale bar, 1 mm. **b**, SEM image of this phototransistor array with 3D liquid microelectrodes (pixel pitch: 40 μm, pillar height: 60 μm, pillar diameter: 5 µm) before depositing the top parylene-C encapsulating layer. Scale bar, 100 μm. This experiment was repeated 2 times independently with similar results. Source data

Although the device used in the in vivo experiment was limited to 36 pixels due to the small mouse eye (diameter, 3 mm), further enlargements in device size and an increase in the number of pixels will enable its application to a large animal model with larger eyeballs and thicker retinas. In addition, our printing system was able to scale down the diameter of the 3D LM microelectrodes to 5 µm. A high-resolution device with 3D LM microelectrodes, which theoretically corresponds to 20/160 vision, can be fabricated (Extended Data Fig. 5)^21^. Reducing the stimulation electrode size is crucial for achieving high-resolution stimulation. However, as the size of the stimulation site decreases, impedance increases, which limits effective stimulation. Further studies on nanoscale materials (for example, Pt nanoclusters), which enhance the stimulation efficacy by adding nanometre-scale roughness to the electrode surface, can be interesting future work for achieving higher visual acuity.

## Methods

### Fabrication of phototransistor arrays for the artificial retina

The device consists of Si channels (340 nm), Cr (5 nm)/Au (100 nm)/Pt (30 nm) source (S)/drain (D)/interconnect electrodes, SiO_2_ dielectric layer (500 nm) and indium tin oxide gate (G) electrode (150 nm). The Si channel, which is a representative photoabsorbing semiconductor, was chosen as a proof of concept, but it can be easily replaced by other alternatives with higher sensitivity and flexibility (that is, two-dimensional materials) to further enhance the optoelectronic performance of artificial retinas. For the fabrication of these phototransistor arrays, first, an array of single-crystalline Si, which serves as the channel of the transistor, was photolithographically patterned using a positive photoresist (S1818, MicroChem) on a silicon-on-insulator wafer (340 nm boron-doped p-type Si with a resistivity of 8.5 Ω cm on 400 nm buried oxide; Soitec). This transistor array was fabricated on a thin and transparent polyimide film (thickness, 8 μm). The Si channels were etched with an RIE system with sulfur hexafluoride (SF_6_) plasma (SF_6_ 25 s.c.c.m./Ar 55 s.c.c.m.; 300 W/40 s), completing the channel isolation process. Any subsequently remaining photoresist residue was removed using a piranha solution (10 min). To separate the Si channel from the silicon-on-insulator wafer, the buried oxide layer was etched in a 50% hydrogen fluoride solution for 18 min. Second, the pattern of Si channels was transferred from a silicon-on-insulator wafer onto the flexible and transparent polyimide film (8 μm) using a polydimethylsiloxane stamp (SYLGARD 184, 10:1 weight ratio of base and curing agent). Cr 5 nm/Au 100 nm were deposited using an electron-beam evaporator and were photolithographically patterned to form a source (S) electrode, a drain (D) electrode and interconnects^41–43^. Then, a sacrificial layer (LOR 3A photoresist, Kayaku) was spun on the substrate and photolithographically patterned, and this was followed by the deposition of 30-nm-thick Pt on the opened area with an electron-beam evaporator. This metal layer was deposited to prevent the penetration of gallium atoms to the Au drain electrodes. Subsequently, silicon dioxide (SiO_2_) was deposited with a thickness of 500 nm at 150 °C using plasma-enhanced chemical vapour deposition, and it was photolithographically patterned as a dielectric layer. Then, for the patterning of the gate (G) electrode, a sacrificial layer (LOR 3A photoresist, Kayaku) was spun on the substrate and photolithographically patterned. Indium tin oxide was deposited as a gate electrode with a thickness of 150 nm at room temperature by radio-frequency magnetron sputtering, and it was immersed in mr-Rem 700 (lift-off solution, micro resist technology) at 60 °C for 30 min to melt the sacrificial layer. As a biocompatible encapsulation layer, a 1-μm-thick layer of parylene C was deposited and photolithographically patterned by dry etching with RIE (O_2_ 40 s.c.c.m., 100 W/240 s) to open the area for the direct printing of 3D LM microelectrodes.

### Fabrication of 3D LM microelectrodes

The key steps in the fabrication of the 3D LM microelectrodes are as follows:

(1) Direct printing of 3D pristine EGaIn electrodes: the direct printing system consists of a capillary nozzle connected to an ink reservoir; a pneumatic pressure controller and a six-axis stage with automatic movements in the *x*, *y* and *z* axes; two tilting axes in the *x* and *y* axes; and rotation in the *x*–*y* plane. First, a pipette puller (P-1000, Sutter Instrument) was used to make a glass capillary (Sutter Instrument) as a nozzle with inner diameters of 5 to 50 μm. Then, a nozzle was mounted onto a syringe-type reservoir, and a substrate was placed on the six-axis stage. All of the LM printing steps were recorded by the microscope camera (QImaging MicroPublisher 5.0 with real-time viewing, Teledyne Photometrics) to control the nozzle from the substrate using the six-axis stage (H-820 6-Axis Hexapod, Physik Instrumente) during the printing process. The distance between the tip of the nozzle and the substrate was controlled to be in the range of 2–16 μm according to the diameter of the nozzle, and the pneumatic pressure (∼50 psi) was applied to deliver the EGaIn ink (75.5% gallium and 24.5% indium alloy by weight; Changsha Santech Materials) from a reservoir onto the substrate through the nozzle. After we controlled the *z* axis of the six-axis stage to make contact between EGaIn and the opened area of the drain electrode, the ink was directly printed in a circular shape on the top surface of the drain to exhibit a thicker base of the 3D micropillar for its structural stability. By adjusting the printing motion along the *z* axis at a velocity in the range of 1 to 500 μm s^–1^, the 3D pillar of EGaIn with a uniform diameter (except the circular base part) can be printed (Supplementary Video 1). On exposure to air, EGaIn instantaneously forms a thin solid layer (∼1 nm) of gallium oxide on its surface under atmospheric oxygen levels to maintain its vertical 3D structure of EGaIn. This oxide skin is thin enough to avoid substantially damaging the cellular interfaces, and it is solid enough to maintain its 3D shape against gravity and surface tension.

(2) Selective opening of 3D electrode tips: after the printing of the 3D pristine EGaIn electrodes, additional parylene C (thickness, 1 μm) was deposited on the entire device, including the 3D electrodes for the passivation of their sidewalls. Only their tips were selectively opened using anisotropic O_2_ RIE (100 W/240 s), as the additional parylene C encapsulating layer served as a protective layer of the first parylene C encapsulation layer as well as the encapsulation layer of the sidewalls of the 3D micropillars.

(3) Deposition of Pt nanoclusters: to prepare 50 ml of an electroplating solution, we mixed 50 ml of deionized water, 10 mg of lead acetate trihydrate (Sigma-Aldrich) and 0.5 g of platinum tetrachloride (Sigma-Aldrich) at room temperature. This electroplating solution was stirred for 20 min by ultrasonic vibration. The electroplating was performed by ion transfer between the cathode and anode in the Pt electroplating solution. After mounting the device to a multichannel recording interface (MZ-60, Tucker-Davis Technologies), a cathode (the 3D pristine EGaIn microelectrode that is to be electroplated) and an anode (Ti/Pt electrode) were immersed in this electroplating solution, and each electrode was connected to a source meter (Keithley 2400, Tektronix). An electrical current of 0.1 mA was applied for 60 s to generate the electroplating reaction (Supplementary Fig. 25). Due to potential variations in currents under light exposure, we performed the electroplating of Pt nanoclusters in the dark state.

(3) Rinsing process of the artificial retina: before the implantation of the device, we rinsed the artificial retina by gently immersing the device in 70% ethanol solution (15 min) and deionized water (15 min) followed by ultraviolet exposure (30 min).

### Ex vivo animal experiments

Ex vivo experiments were conducted based on the guidelines and were approved by the Institute of Animal Care and Use Committee of Yonsei University (IACUC-A-201911-985-01, IACUC-202011-1164-05, Yonsei IACUC). The recording involved the retinas of five mice for both WT and rd1 type, and for the recording of retinal responses (that is, visually or electrically evoked retinal spike potentials and firing rate of the spikes); each recording electrode was positioned adjacent to each stimulation electrode (pitch between the stimulating and recording electrodes, 40 μm). The retinas of both WT mice (C57BL/6J, Japan SLC) and rd1 mice (C3H/HeJ, Japan SLC) were explanted, and small pieces (∼4 × 4 mm) were isolated and transferred to the artificial retina with the phosphate-buffered saline medium by RGCs facing the device, and a heating pad was used to maintain the temperature of the retinas at 37 °C. The animal was immediately sacrificed after extraction. The isolated retinas from WT and rd1 mice were directly placed on our device (consisting of 36 stimulating and recording electrode pairs), and our 3D electrodes were directed towards the RGC side of the retina in phosphate-buffered saline media. Immediately before implanting this device into the retina, the device sample was instantly frozen to turn the liquid-phase EGaIn into a solid by leaving it in cold storage (below the melting point of EGaIn, ∼15.7 °C). Then, the protrudent pillar shape of the 3D electrodes returned to a liquid phase and did not collapse even after being implanted into the retina.

Since mice are dichromatic mammals having only two cone types (blue and green light sensitive)^42,43^, the blue light (wavelength, 470 nm) was used for exposure in this experiment. For electrical stimulation, the transistor of our device was operated with a specific condition (*V*_G_, d.c. bias of 5 V; *V*_D_: pulsed bias of 1 V with a duration of 1 ms and frequency of 10 Hz) and the recordings were performed with the adjacent recording electrodes. Electrophysiological recordings of the retina were conducted by multielectrode array recording and multichannel stimulation (PZ5 and Subject Interface, Tucker-Davis Technologies) and a data processor with a real-time controller (RZ2 BioAmp Processor, Tucker-Davis Technologies). We recorded the VEP and EEP signals at a 25 kHz sampling rate using a 300 Hz low-pass filter and 3,000 Hz high-pass filter. The experimental data were processed further by applying a band-pass filter with MATLAB R2021a (MathWorks). No data points were excluded from the analyses.

### In vivo implantation

The rd1 mice (*n* = 3) were anaesthetized with an intraperitoneal injection of a mixture of tiletamine and zolazepam (1:1, 15 mg kg^–1^ body weight) and xylazine hydrochloride (10 mg kg^–1^ body weight). The pupils of the mice were dilated with eye drops that contained 0.5% phenylephrine and 0.5% tropicamide. The body temperatures of the mice were maintained at 37 °C with a heating pad.

For the surgical procedures, the mouse was placed in a head holder to maintain the head in a fixed position and to allow access to the eye. The head holder was placed under an optical microscope with an illuminator. A clear 2.2 mm corneal knife (KAI MEDICAL; CCR-22AGF) was used to make a 1.5 mm incision in the area of the pars plana. Immediately before implanting this device into the retina, the device sample was instantly frozen to turn the liquid-phase EGaIn into a solid by leaving it in cold storage (below the melting point of EGaIn, that is, ∼15.7 °C). Then, it was implanted into the vitreous cavity (that is, attached to the retinal surfaces) via the incision that was made earlier. For preventing cataracts during a continuous functional analysis, a 10 g drop of hypromellose (Hycell oph soln) was applied to the surface of the cornea. During this in vivo experiment, a hydrogel-based artificial vitreous body was initially filled in the vitreous cavity of the mouse eye to prevent undesired side effects, such as hypotony (low intraocular pressure). After the experiment, the mice were immediately euthanized by carbon dioxide inhalation in a carbon dioxide chamber. No statistical methods were used to predetermine the sample sizes, but our sample sizes are similar to those reported in previous publications^9,11^.

### In vivo animal experiments

In vivo experiments were conducted based on the guidelines of the Institute of Animal Care and Use Committee of Yonsei University (IACUC-A-202205-1478-01, Yonsei IACUC). Considering the size of the eyeball of a mouse (diameter, ∼3 mm), we fabricated an artificial retina integrated with 6 × 6 arrays of phototransistors (pixel pitch, 200 μm; device width, 2 mm) with 3D LM microelectrodes (height, 60 μm; diameter, 20 μm). This artificial retina was implanted into the innermost retinal surface of the rd1 mouse epiretinal, with external device interconnections. All the devices and animals tested were randomly selected. The recording lines were connected to the glass pad with interconnect electrodes and then patterned with photolithography and wet etching after the deposition of Cr/Au (10/100 nm) by an electron-beam evaporator. The interconnect pad was inserted into the multielectrode array recorder with a multichannel stimulator (PZ5 and Subject Interface, Tucker-Davis Technologies) and a data processor with a real-time controller (RZ2 BioAmp Processor, Tucker-Davis Technologies). The multichannel experimental data of the spike signal and firing rate were obtained and exported by analysis software (Synapse Suite version 94, Tucker-Davis Technologies). Then, the data were processed and mapped with MATLAB R2021a (MathWorks) and Origin 2022b software. Full-field light illumination (470 nm, TouchBright T1 with BN470 band-pass filter, Live Cell Instrument) or a laser (wavelength, 415 nm) through an ellipsoidal pattern of a shadow mask was applied to the fundus of the mouse’s eye for light exposure (duration, 5 s). No data points were excluded from the analyses. Also, data met the assumptions of the statistical tests used.

### Statistical analysis

All data were presented as mean ± standard deviation (s.d.). Statistical calculations of *P* value were performed using an open-source code of MATLAB R2021a. Significance was calculated using an unpaired one-tailed *t*-test.

### Reporting summary

Further information on research design is available in the Nature Portfolio Reporting Summary linked to this article.

## Online content

Any methods, additional references, Nature Portfolio reporting summaries, source data, extended data, supplementary information, acknowledgements, peer review information; details of author contributions and competing interests; and statements of data and code availability are available at 10.1038/s41565-023-01587-w.

### Supplementary information

Supplementary Information Supplementary Figs. 1–26, Table 1 and Methods.

Reporting Summary

Supplementary Table 1 Thickness of the retinal layers of the WT mice retina.

Supplementary Video 1 Real-time demonstration of 3D EGaIn pillar using the direct printing method.

### Source data

Source Data Fig. 1 Statistical source data.

Source Data Fig. 2 Statistical source data.

Source Data Fig. 3 Statistical source data.

Source Data Fig. 5 Statistical source data.

Source Data Extended Data Fig./Table 1 SEM images.

Source Data Extended Data Fig./Table 5 Photograph and SEM images.

## Data Availability

The data regarding the characterization of the artificial retina and animal experiments are available via Figshare at 10.6084/m9.figshare.22815461. Statistical source data are provided with this paper. The raw datasets generated during this study are available from the corresponding authors upon reasonable request. Source data are provided with this paper.

## Code availability

The custom codes for MATLAB used in this study are available from the corresponding authors upon reasonable request.
