# Supplementary material for: Liquid-metal-based three-dimensional microelectrode arrays integrated with implantable ultrathin retinal prosthesis for vision restoration
Source: Nat Nanotechnol. 2024 Jan 15;19(5):688–97. doi: 10.1038/s41565-023-01587-w (PMC11106006; doi:10.1038/s41565-023-01587-w)
Supplement: Supplementary file 1 — Supplementary Figs. 1–26, Table 1 and Methods. [file 41565_2023_1587_MOESM1_ESM.pdf]

nature nanotechnology

Article https://doi.org/10.1038/s41565-023-01587-w

Liquid-metal-based three-dimensional microelectrode arrays integrated with implantable ultrathin retinal prosthesis for vision restoration

In the format provided by the authors and unedited

This supplementary information file includes:

Supplementary Methods

Supplementary Figures 1 to 26

Other supplementary files for this manuscript includes:

Supplementary Table 1

Supplementary Video 1

Supplementary Methods

Mobility calculation

Mobility of the Si channel FET was calculated using the equations of the standard metal-oxide-semiconductor FET model (shown below):

$$\Delta I_D = \frac{\mu \times W \times C_i \times V_D \times \Delta V_G}{L} \rightarrow \mu = \frac{L \times {}^{\Delta I_D}/_{\Delta V_g}}{W \times C_i \times V_D} = \frac{L \times g_m}{W \times C_i \times V_D}$$

Electrical characterization of Si phototransistor arrays

The transfer and output characteristics of transistors were determined by using a probe station (Keithley 4200-SCS). Blue light was illuminated with different intensities on the phototransistors to measure their photo-responsive properties.

Characterization of the optoelectronic properties of the artificial retina

For this experiment, an eagle-patterned mask was fabricated to locally block the light by the electron-beam (e-beam) deposition of Cr/Au (10/100 nm) on a 25 μm-thick transparent PET film. Then photolithography and wet etching were used to pattern the eagle-patterned shadow mask. After placing this mask directly on the phototransistor array, the photocurrent (I_D) induced by the local light penetration (light intensity: 1.80 mW cm^{-2}, wavelength of 470 nm, TouchBright T1 with a BN470 bandpass filter, Live Cell Instrument, USA) was mapped spatially under the illumination by visible light. The light intensity was measured with an LX1330B digital light meter (Dr.meter, USA). The photocurrents were measured using real-time transistor switching systems consisting of two sourcemeters (Keithley 2400), a system switch (Keithley 3706), and a relay card (Keithley 3723).

Characterization of the electrochemical properties of the 3D liquid microelectrodes

The electrochemical properties (i.e., the impedance and current-voltage characteristics) of the 3D liquid microelectrodes were measured by using electrochemical impedance spectroscopy (EIS) and cyclic voltammetry (CV) analysis with a multichannel potentiostat (Parstat MC-1000, AMETEK, USA) to compare the impedance and CSC characteristics of these 3D electrodes. All of the electrodes were immersed in a phosphate-buffered saline (PBS) solution (pH 7.4) at ambient conditions, and the Ag/AgCl electrode and Pt sheet electrode were used as a reference and counter electrode, respectively. Impedance was measured by sweeping the frequencies from 10 Hz to 100 kHz, and the characteristics of the CV were measured at a scan rate of 50 mV s^{-1} with potential limit ranges from -0.6 V to 0.8 V.

Cell viability test

1) **Live/dead cell assay:** A live/dead cell assay test was conducted with human retinal pigmented epithelium (RPE) cells (Human ARPE19 cells, ATCC, Wesel, Germany). DAPI staining (NucBlue® Live ReadyProbes® Reagent Protocol, R37605, Life Technologies, Grand Island, NY) and live/dead cell assay was made (Live/dead cell imaging kit 488/570, R37601, Molecular Probes, Life Technologies Corp., CA, USA) for reference, positive control (cells with G418), negative control (PI film), and the artificial retina, respectively. An EVOS® fl digital inverted fluorescence microscope (Advanced Microscopy Group, USA) was used to examine the cell viability of live and dead human RPE cells between the device and the reference.

2) **Flow cytometry:** Apoptosis assay was conducted with human RPE cells (Human ARPE19 cells, ATCC, Wesel, Germany). The artificial retina device and PI film were each cut into 2 mm x 1.5 mm pieces and attached to a 96-well cell culture plate. Human RPE cells were seeded with 3 x 10^3 on the reference, negative control (PI film), positive control (puromycin-treated cells), and artificial retina and cultured at 37 °C, 5% CO_2 for 7 days. The positive control was treated with puromycin at a concentration of 0.5 µg ml^{-1}. To harvest cultured cells on artificial retinal devices and PI films, the devices and films were detached with forceps, transferred to 1.5 ml tubes, and treated with 0.25%

trypsin/EDTA (T/E). The reference and puromycin-treated positive controls were harvested by treating the cells on the plates with 0.25% T/E. Harvested cells were double stained using the Annexin V conjugated with fluorescein isothiocyanate (FITC) and propidium iodide in the kit (Annexin V-FITC Apoptosis Detection kit, APOPF, Sigma-Aldrich, MO, USA). Annexin V conjugated with FITC labels phosphatidylserine sites that become exposed on the surface of the cell membrane as apoptosis progresses. Propidium iodide was used to label DNA from necrotic cells, where the cell membrane is completely damaged. To distinguish between live cells (Annexin V-FITC negative, PI negative), necrotic cells (Annexin V-FITC positive, PI negative), and early apoptotic cells (Annexin V-FITC positive, PI negative), they were analyzed by flow cytometry (BD FACS Verse, Becton Dickinson and company, NJ, USA). Flow cytometry was performed with the following gating strategy. The data was evaluated by two researchers who were blinded to the information.

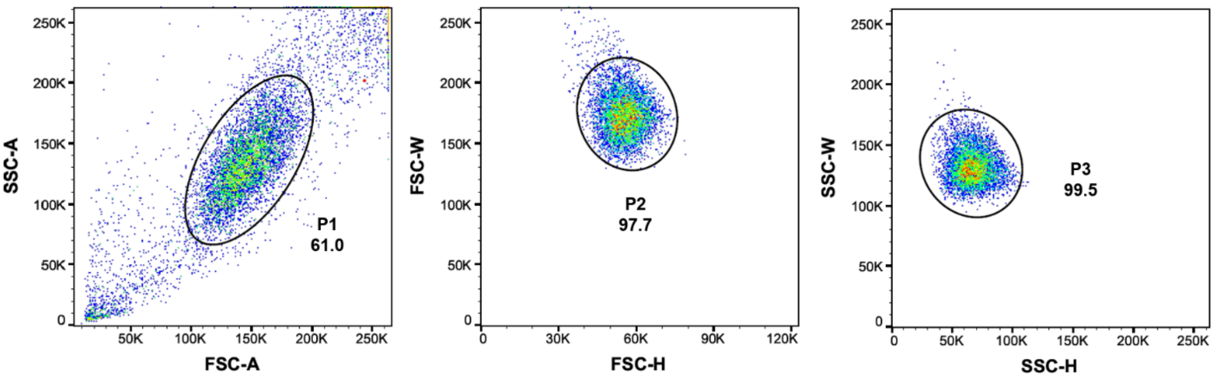

Immunohistochemistry

To analyze cross-sectional images of the mouse retina and device, the eye was enucleated and fixed for 1 hour in 4% paraformaldehyde in PBS. The cornea and lens were removed, and the remaining eye cups and devices were soaked in 15% sucrose in PBS, followed by equilibration in 30% sucrose. The eye cups were then embedded in OCT compound and snap-frozen in liquid nitrogen. Samples were cut into 30 µm thick sections using a cryostat. Cryosections of the mouse retina were immunostained with primary antibodies: anti-CD68 antibody (Cell Signaling Technology, USA), anti-

CD11b antibody (Abcam, USA), and anti-SNCG antibody (Abnova, Taiwan). Secondary antibodies were also used.

For mouse retina flat-mount staining, enucleated mouse eyes were fixed for 1 hour in 4% paraformaldehyde in PBS. The anterior chamber and lens were removed. While being careful not to touch the device and the area in contact with the retina, the eyes were cut into a four-petal pattern. After incubating with primary and secondary antibodies, the eyecups were flat-mounted on a slide.

All images were generated using confocal microscopy (Zeiss LSM 700) and edited using ZEN 2011 software.

Tensile tests

The elastic modulus was measured by stress-strain characteristics during a tensile test using a TXATM micro-precision texture analyzer (Yeonjin S-Tech, Republic of Korea). The strain rate was 10 μm/s during its stretching and releasing.

Fabrication of an artificial retina for *ex-vivo* experiments

For the fabrication of the device for *ex-vivo* experiments, the same process was used that was used for the artificial retina. An array of single-crystalline Si, which serves as the channel of the transistor, was patterned photolithographically using a positive photoresist (S1818, MicroChem) on an SOI wafer (silicon-on-insulator, 340 nm Si on 400 nm buried oxide, Soitec, France) diced into a 5 x 5 cm^2 square-shaped device. The Si channels were etched with a reactive ion etching (RIE) system with sulfur hexafluoride (SF_6) plasma (SF_6 25 SCCM / Ar 55 SCCM, 300 W / 40 sec), completing the channel isolation process. Any remaining photoresist residue was removed subsequently by the Piranha solution (10 min). The pattern of Si channels was transferred from a silicon-on-insulator (SOI) wafer onto the flexible and transparent polyimide (PI) film (8 μm) using a polydimethylsiloxane (PDMS) stamp (Sylgard 184, 10:1 weight ratio of base and curing agent). Then, Cr 5 nm/Au 100 nm

were deposited using an e-beam evaporator directly on the etched SOI wafer, and they were patterned photolithographically in order to form the source (S) electrode, the drain (D) electrode, and the interconnects. Then, a sacrificial layer (LOR 3A photoresist, Kayaku) was spun on the substrate and was photolithographically patterned followed by the deposition of a 30 nm thickness of Pt on the opened area with an e-beam evaporator. This metal layer was deposited to prevent the penetration of gallium atoms to the Au drain electrodes. Subsequently, silicon dioxide (SiO_2) was deposited with a thickness of 500 nm at 150 °C using plasma-enhanced chemical vapor deposition (PECVD), and it was patterned photolithographically as a dielectric layer. Then, for the patterning of the gate (G) electrode, a sacrificial layer (LOR 3A photoresist, Kayaku) was spun on the substrate and photolithographically patterned. Indium tin oxide (ITO) was deposited as a gate electrode with a thickness of 150 nm at room temperature by radio frequency (RF) magnetron sputtering, and the ITO was immersed in mr-Rem 700 (Lift-off solution, Micro resist technology) at 60 °C for 30 minutes to melt the sacrificial layer. As a biocompatible encapsulation layer, a 1-μm-thick layer of parylene-C was deposited and was photolithographically patterned by dry etching with RIE (O_2 40 SCCM, 100 W / 240 sec) to open the area for direct printing of the 3D liquid microelectrodes. Then, the polydimethylsiloxane (PDMS) layer was perforated by a laser ablation machine to serve as a microwell. The phototransistor arrays with 3D liquid microelectrodes were placed in the vacant hole of this PDMS microwell to promote the immersion and the localization of the retina in the PBS medium during the electrophysiological recording.

***Ex-vivo* data analysis**

The refined retina signals from the *ex-vivo* evaluation were obtained by the clustering algorithm, which is an unsupervised machine learning approach[1]. For the primary categorization, hierarchical clustering was conducted to distinguish the natural criterion of the magnitude and shape of the complex signal data. Hierarchical clustering consists of building a dendrogram, starting from

the data sets and proceeding by merging two by two the closest data sets or clusters until all of the data sets are merged to one cluster[1,2]. The distance between two different data sets or clusters is defined by Euclidean distance as follows:

$$d(p,q) = \sqrt{\sum_{i=1}^{n}(q_i - p_i)^2} \tag{1}$$

where $d(p,q)$ is the Euclidean distance between two data points $p = (p_1, p_2, \ldots, p_n)$ and $q = (q_1, q_2, \ldots, q_n)$, and n is the dimension of the data sets. The closest data sets or clusters were defined by Ward's minimum variance method, where the pair of clusters that leads to a minimum increase in the objective function is found after merging them and chosen as an upper cluster[3]. The objective function was used as the sum of squares error (SSE) as follows:

$$SSE_i = \sum_{j=1}^{n_i}\sum_{k=1}^{m}(p_{ijk} - \bar{p}_{ik})^2 = \sum_{j=1}^{n_i} d(p_{ij}, \bar{p}_i) \tag{2}$$

where n is the number of data sets, m is the dimension of the data sets, and $\bar{p}_{ijk}$ is the averaged distance of the data point $p_{ijk} = (p_{ij1}, p_{ij2} \ldots, p_{ijk})$. The sizes and shapes of 4,992 initial data sets of retina spikes were evaluated by the hierarchically classified clusters and sorted out to the most relevant 2,880 retina spikes. The input values for clustering were the absolute value of the signals at each time in the *ex-vivo* evaluation, and each measurement time was set to variables of the input values.

Next, the hierarchically classified 2,880 retina spikes went through an additional K-means clustering process. K-means clustering is to classify the given data sets into K different clusters by minimizing the SSE of Euclidean distances among K different clusters' centers[4,5]. The number of cluster K is a hyperparameter, and it was predetermined by the Elbow method and the Silhouette method[6,7]. The Elbow method calculates the within-cluster sum of squares (wcss) by increasing K, and it suggests the optimal K when the wcss changes dramatically. The Silhouette method suggests the optimal K by calculating the silhouette coefficient, which is described as follows:

$$s^{(i)} = \frac{b^{(i)} - a^{(i)}}{\max\{a^{(i)} b^{(i)}\}} \tag{3}$$

where $s^{(i)}$ is the silhouette coefficient of the *i*-th data set, $a^{(i)}$ is the average distance between the *i*-th

data set and other data sets in the same cluster, and $b^{(i)}$ is the average distance between the i-th data set and other data sets in a different cluster. The terms $a^{(i)}$ and $b^{(i)}$ represent the cohesion and separation of the clusters, respectively. The optimal K is defined when the silhouette coefficient is closest to 1. Using both methods, the optimal K was selected as 4 for retina spikes clustering, and 2,880 retina spikes were classified into four different clusters. Python (ver. 3.7.6) and Scikit-Learn library (ver. 0.22.1) were used for clustering algorithms for the machine learning approach.

Histological analysis

Eyes were fixed on Davidson's Fixative solution (Sigma; catalog H0290-500ML) for 24 hours before paraffin embedment. Paraffin blocks were sectioned at a thickness of 5 μm and stained with hematoxylin and eosin. All images were obtained using the fluorescent upright microscope (Olympus; BX43).

***In-vivo* data analysis**

In-vivo neural signals were recorded with 300 Hz high-pass filters and 5 kHz low-pass filters using a data processor with a real-time controller (RZ2 BioAmp Processor, Tucker-Davis Technologies, USA) and signal acquisition software (Synapse, Tucker-Davis Technologies, USA). The firing rates were calculated with stimulus-time histograms in OpenExplorer (Tucker-Davis Technologies, USA).

OCT imaging

For the imaging of optical coherence tomography, Image-Guided 830 nm OCT for the Phoenix Retinal Imaging System was used. First, the focus knob was positioned at the middle of the Micron III. Second, the mouse was placed in front of the OCT so that its cornea was positioned directly in front of the tip of the lens and close enough to be in focus in the StreamPix image. Third, adjustment

to the illumination control was made to see the clear fundus image. Last, OCT scanning started when the retina layer came into focus. The bright field fundus image and OCT scan image were captured by StreamPix 5 software and Micron OCT software, respectively. To obtain clear cross-sectional OCT image, only the 3D liquid microelectrodes (height of 60 μm, diameter of 20 μm) formed on a 8 μm-thick PI film were implanted to the retinal surface, to prevent image distortion due to the reflections of incident light to other device components (i.e. transistors and other interconnect metals).

Stability evaluation of 3D liquid-metal microelectrodes

To investigate the collapse of 3D liquid-metal microelectrodes after implantation, the tilted angle of pillars from OCT images after implanting a total of 180 3D microelectrodes to the retina using five rd1 mice (36 pixels per device). The bright field fundus image and OCT scan image were captured by StreamPix 5 software and Micron OCT software, respectively.

We also demonstrated (i) 6 × 3 3D microelectrodes and (ii) 6 × 2 planar lines (similar to the collapsed shape of the 3D pillars) on an 8 μm-thick PI film (Supplementary Fig. 26a and 26b). To clearly identify the shape of these electrodes through the substrate and fundus, these printed electrodes were not interconnected with transistors and were only encapsulated using a parylene-C layer (thickness: 1 μm). After implanting both these 3D and planar electrodes on the retinal surface, Supplementary Fig. 26c shows the line shape of the planar electrodes, in contrast to the dot shape of the vertical 3D electrodes. These 3D microelectrodes distinctively retained their pillar-like protruding structure even after implantation. The printing steps were recorded by the microscope camera (Qimaging Micropublisher 5.0 RTV, Teledyne Photometrics). The fundus image was captured by StreamPix 5 software.

Supplementary Figures

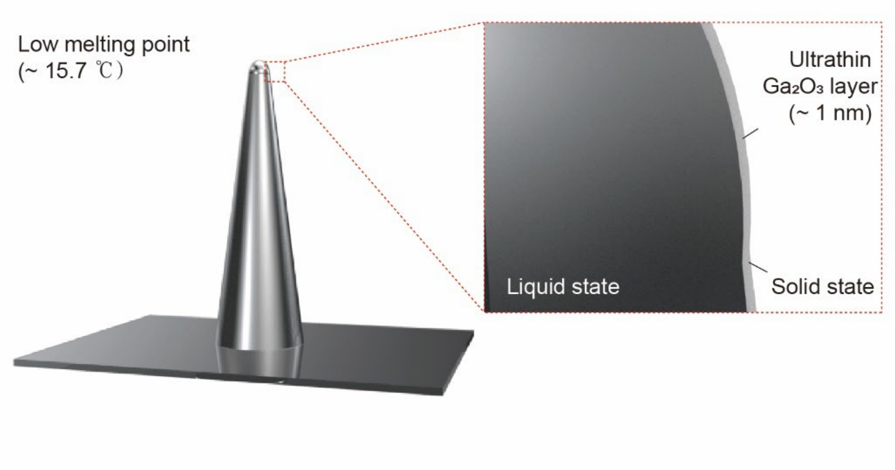

Supplementary Figure 1. Schematic illustration on a 3D-printed pillar pattern of EGaIn. Upon exposure to air during their 3D printing process, these gallium-based metals intrinsically form an ultrathin solid layer of native gallium oxide (thickness: ~1 nm) on their surfaces under atmospheric oxygen levels, and the ultrathin solid oxide skin allows them to maintain 3D shapes of these liquid metals against gravity and surface tension.

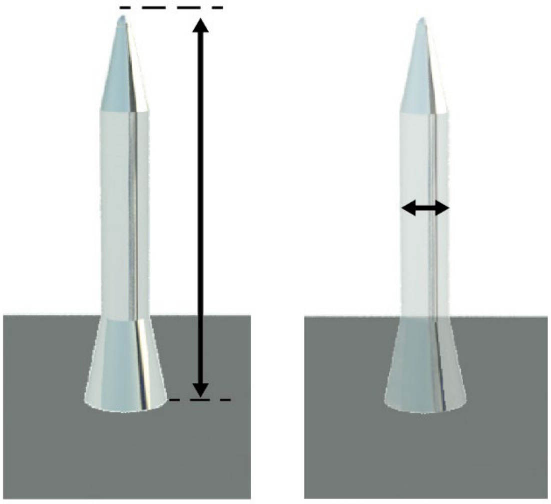

Supplementary Figure 2. Schematic illustrations defining the height and diameter of a 3D-printed pillar. The diameter of the pillar was measured at the midpoint of the height.

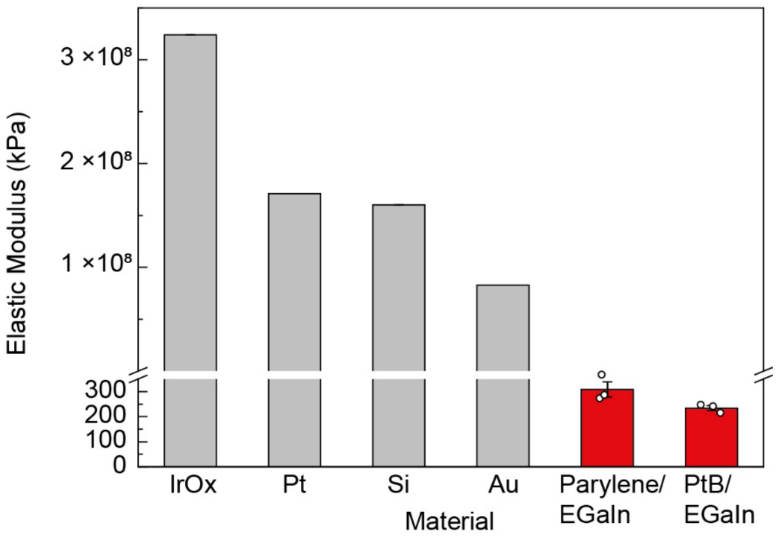

Supplementary Figure 3. Modulus values for various materials as neural interfacing electrodes. Red bar presents a modulus of 3D liquid-metal microelectrode sidewalls (Parylene/EGaIn, 314 kPa) and the tips (PtB/EGaIn, 234 kPa). Data are mean ± S.D. with n = 3 independent experiments. The circles indicate individual data points.

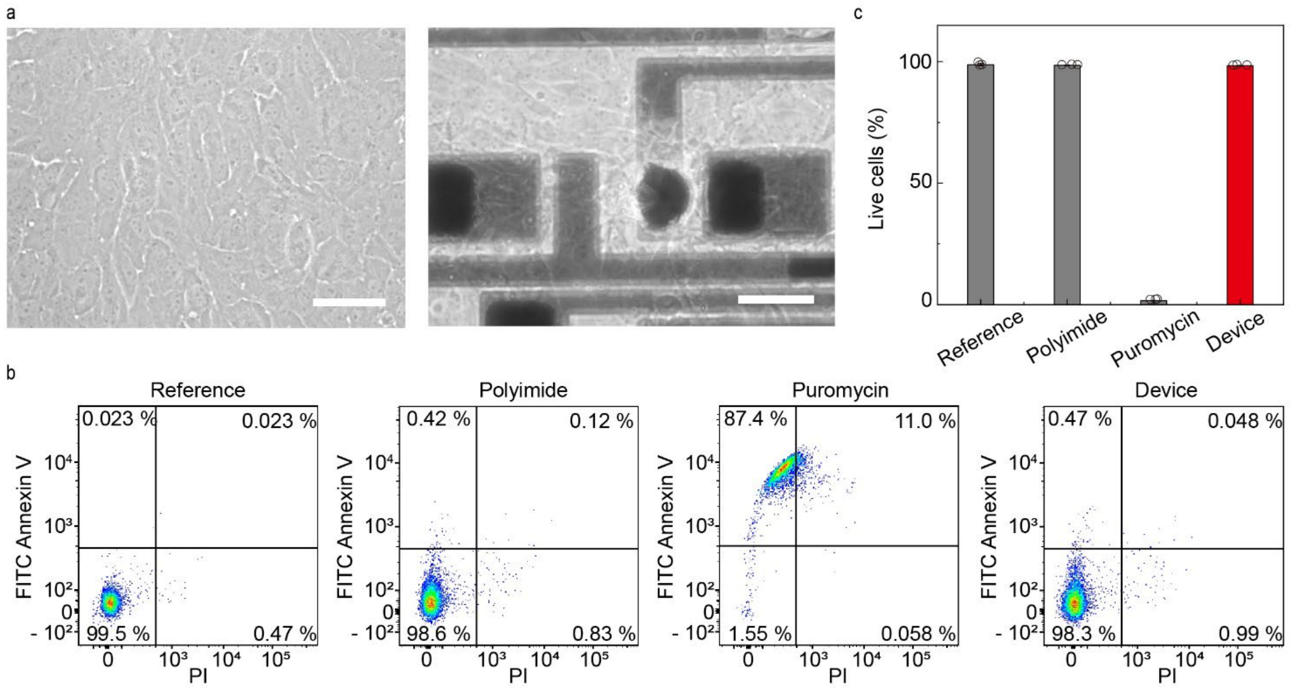

Supplementary Figure 4. *In-vitro* apoptosis assay. **a,** Representative phase-contrast microscopic images of human RPE cells cultured on the device with 3D microelectrodes and on the reference for 7 days, respectively. Scale bars, 50 μm. This experiment was repeated 3 times independently with similar results. **b,** Flow cytometry analysis using Annexin V-FITC and propidium iodide (PI) of cells cultured on reference, polyimide, positive control (cells with puromycin), and device with 3D microelectrodes. **c,** Live cells percentage of the result of **b**. The red bar presents the live cells percentage of the device with 3D microelectrodes. Data are mean ± S.D. with n = 3 independent experiments. The circles indicate individual data points.

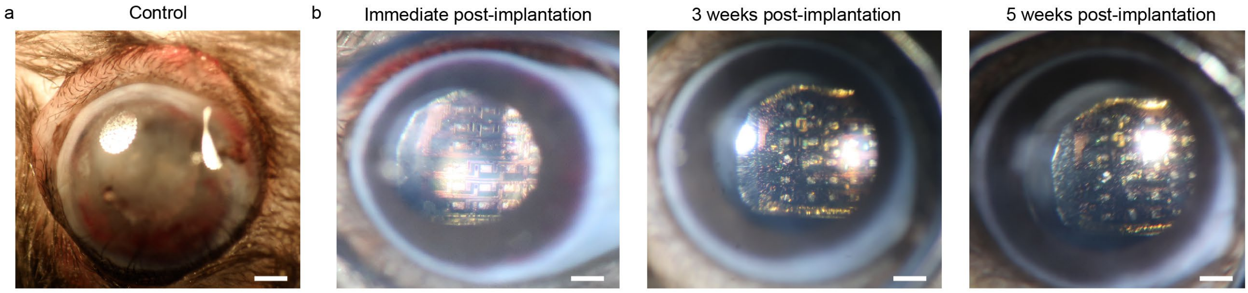

Supplementary Figure 5. a, Fundus image of a live rd1 mouse exhibiting corneal degeneration and cataracts resulting from drug-induced ocular toxicity with no implantation of our device. **b,** Fundus images of the rd1 mouse 5 weeks post-implantation. Scale bars, 500 μm.

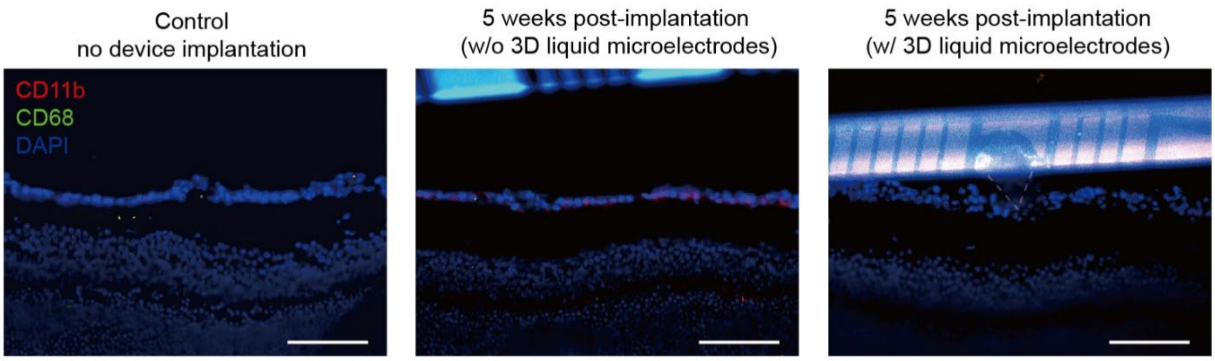

Supplementary Figure 6. Representative immunofluorescence of retinal sections showing the expression pattern of CD11b and CD68 following implantation. CD11b (Red): Microglia cell; CD68 (Green): Macrophage cell, DAPI (Blue): Nucleus. Scale bars, 100 μm. The dotted lines represent the 3D microelectrodes. This experiment was repeated 3 times independently with similar results.

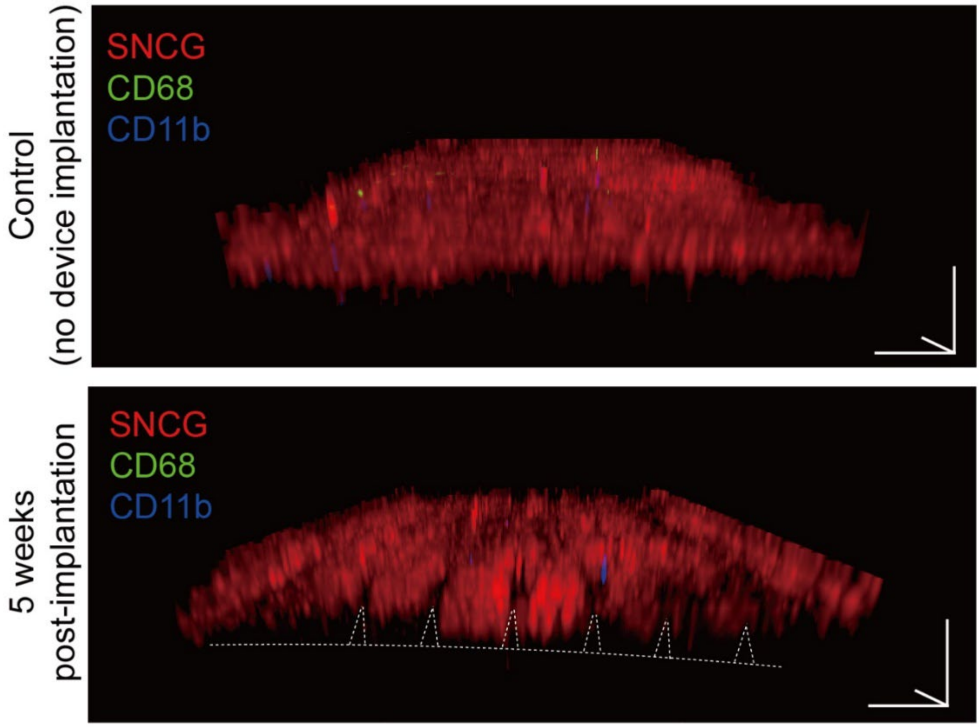

Supplementary Figure 7. 3D-rendered images of whole-mount mouse retina, immunostained with SNCG, CD68, and CD11b antibodies. SNCG (Red): Ganglion cells; CD68 (Green): Macrophage cells; CD11b (Blue): Microglia cells. The dotted lines represent the 3D microelectrodes. Scale bars, 200 µm.

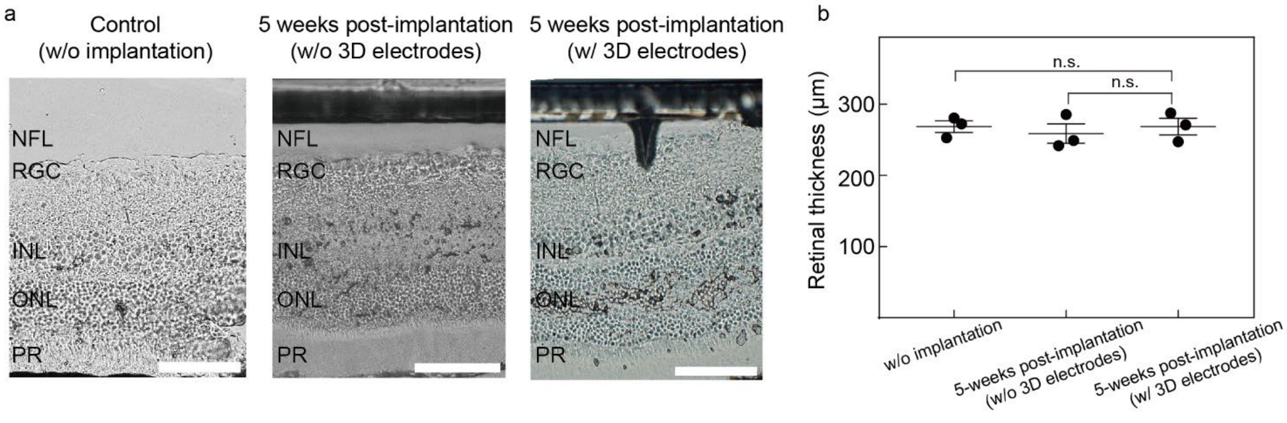

Supplementary Figure 8. a, Bright-field images of retinal sections showing the pillar targeting retinal ganglion cells without collapsing. Scale bars, 100 μm. **b,** Direct measurements of thickness of the retina with implanted devices. Data are mean ± S.D. with n = 3 independent experiments for each group. Statistical analysis was performed using one-way ANOVA for three groups.

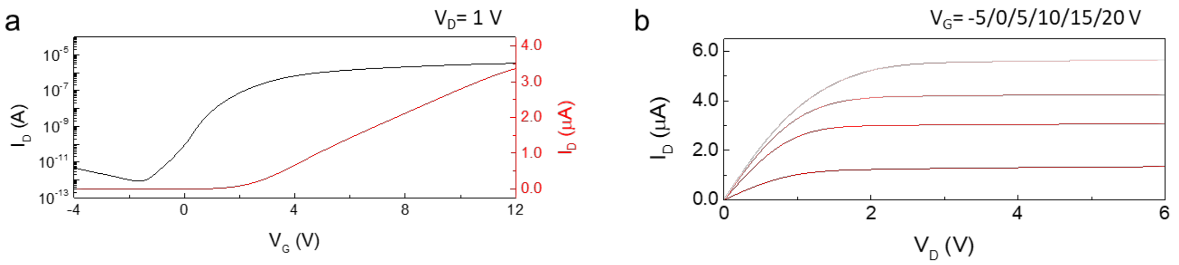

Supplementary Figure 9. I-V characteristic of Si phototransistor. **a,** Transfer characteristics and **b,** output characteristics.

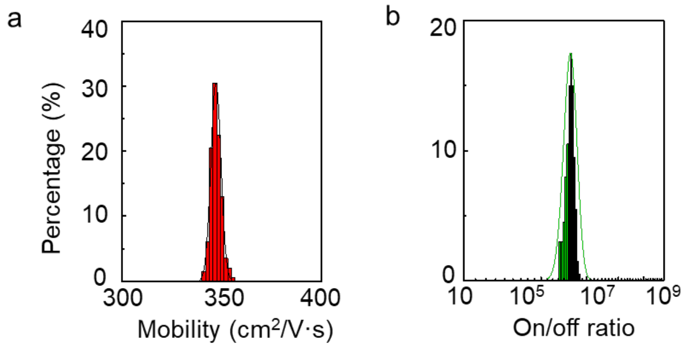

Supplementary Figure 10. The statistical analysis on the **a,** field-effect mobility; **b,** on/off ratio; and **c,** threshold voltage of 2,500 Si phototransistors in total.

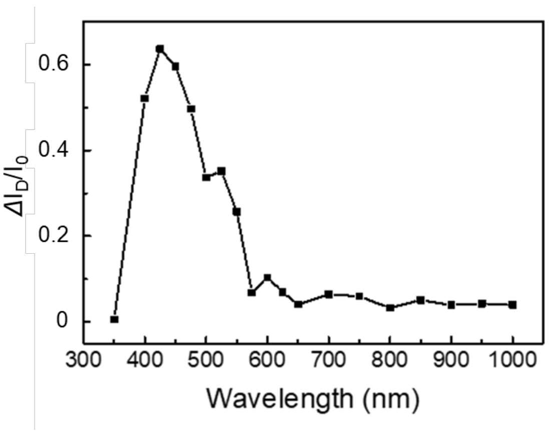

Supplementary Figure 11. Relative changes in I_D of Si phototransistors as a function of light wavelength ranging from 350 nm to 1,000 nm (V_D = 1 V, V_G = 5 V, Light intensity = 1.8 mW cm^{-2}).

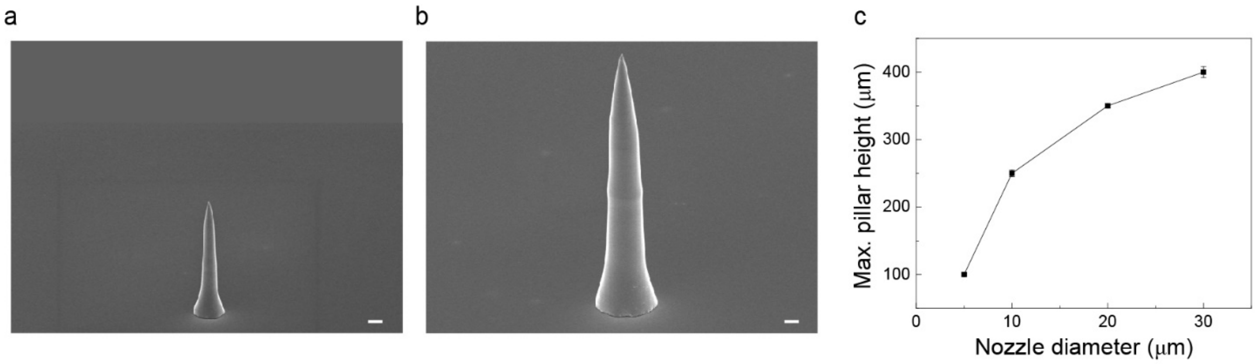

Supplementary Figure 12. a, SEM image of a pillar (diameter: 5 μm). Scale bar, 5 μm. **b,** SEM image of a pillar (diameter: 30 μm). Scale bar, 10 μm. **c,** Plot on the maximum pillar height as a function of nozzle diameter.

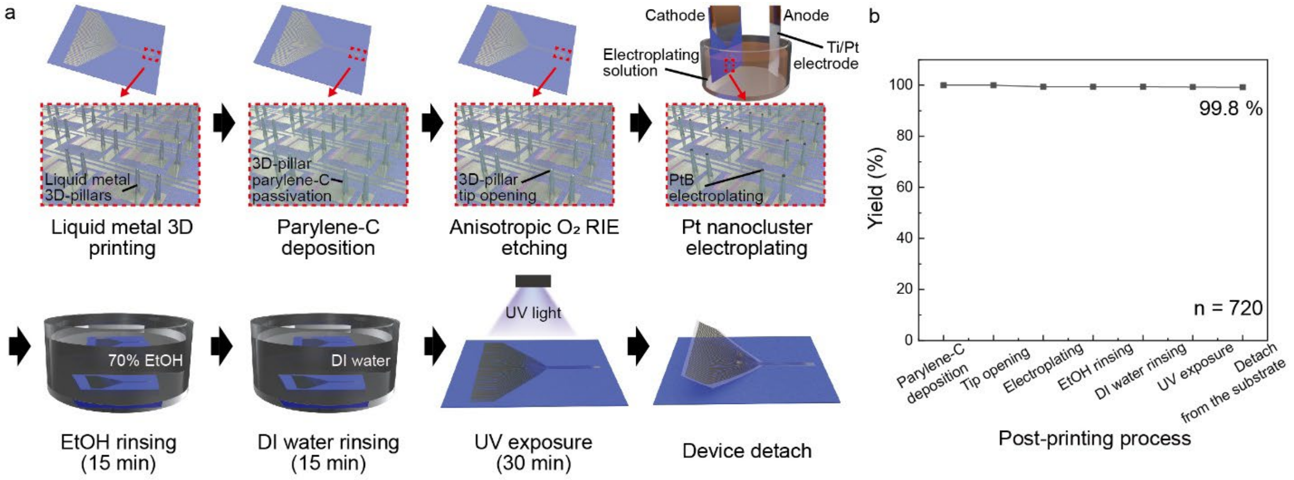

Supplementary Figure 13. a, Schematic illustrations on the process after printing EGaIn pillars. **b,** Yield of the undamaged 3D microelectrodes following each process after printing EGaIn pillars.

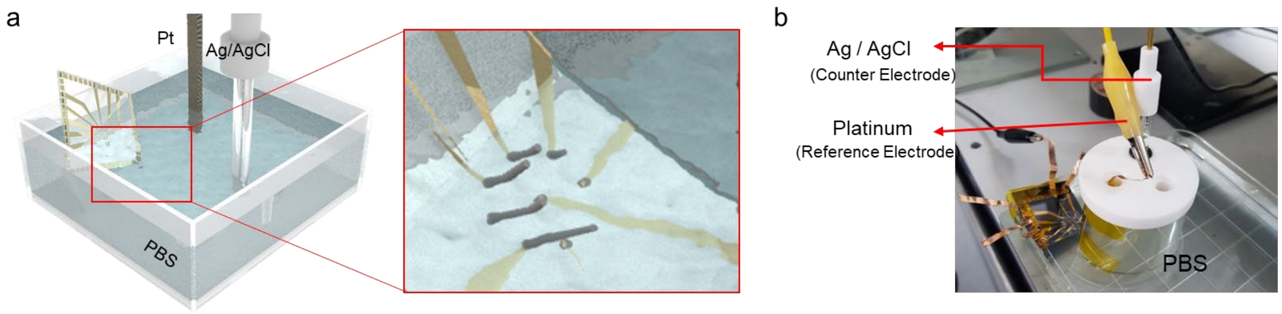

Supplementary Figure 14. a, Schematic illustrations and **b,** photograph of the experimental procedure for the impedance and charge density measurement.

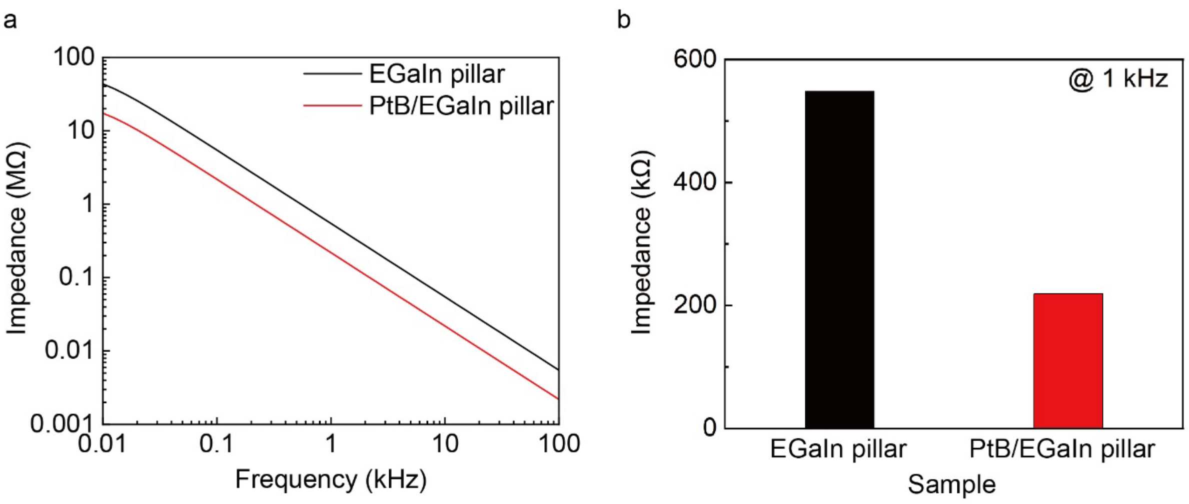

Supplementary Figure 15. a, Impedance spectroscopy of pristine EGaIn pillar and PtB-coated electrode with the same geometry and parylene surrounding (diameter of 20 μm and height of 60 μm). **b,** Comparison of the impedance at 1 kHz of pristine EGaIn pillar and PtB-coated electrode with the same geometry and parylene surrounding (diameter of 20 μm and height of 60 μm, geometrical surface area: 25 μm^2).

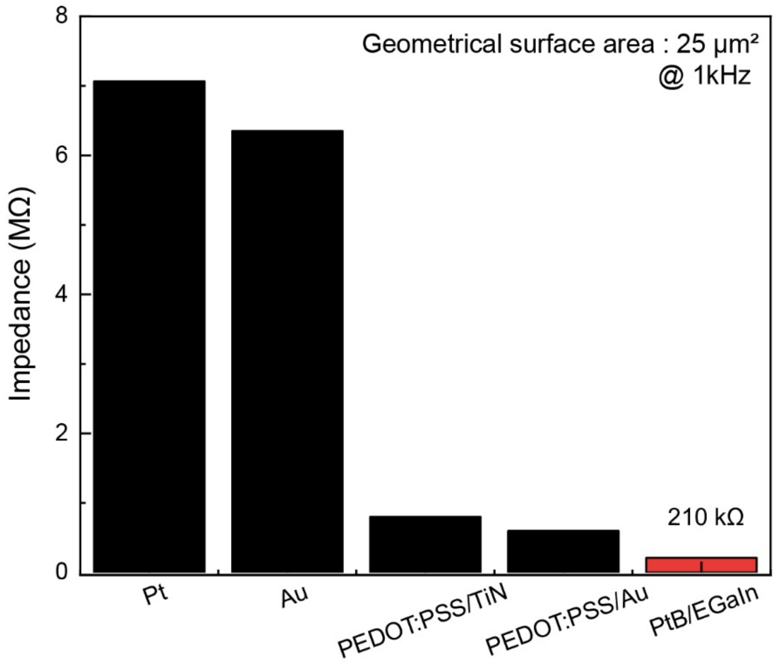

Supplementary Figure 16. Comparison of the impedance values (at 1 kHz) of various materials used for neural interfaces (geometrical surface area: 25 μm^2)[8].

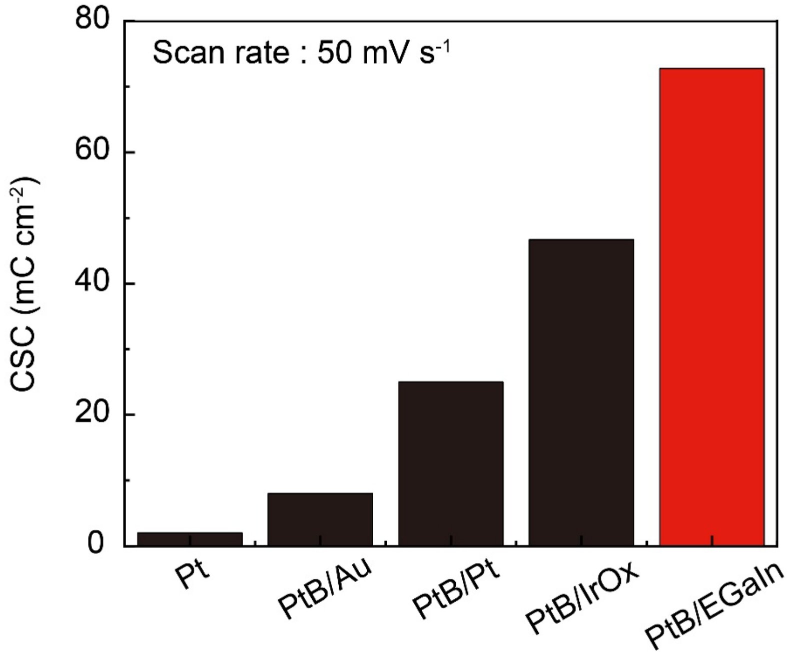

Supplementary Figure 17. Comparison of the charge storage capacities of PtB-coated EGaIn electrode and other typical materials (Scan rate: 50 mV s^{-1})[9,10].

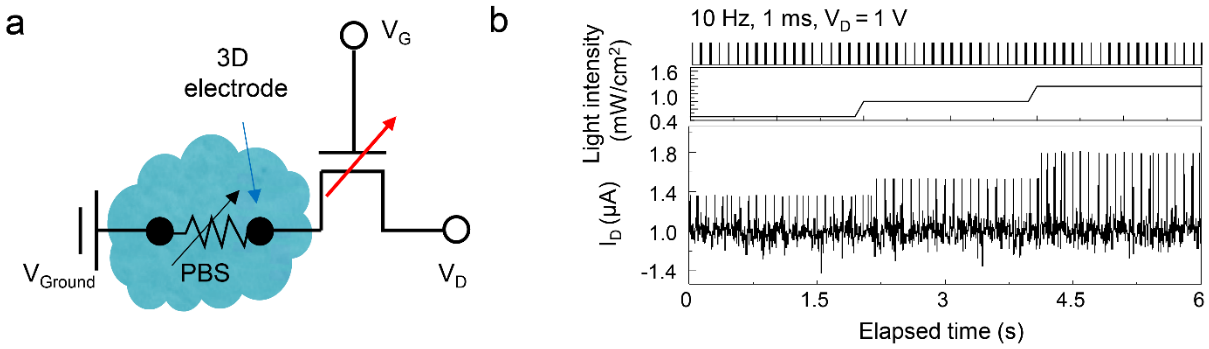

Supplementary Figure 18. a, Circuit diagram of experimental set-up for the stimulation electrode evaluation, **b,** changes in I_D under the various intensities of light illumination under the pulsed bias of V_D. This artificial retina sample was immersed in a PBS medium for the characterization of stimulation,

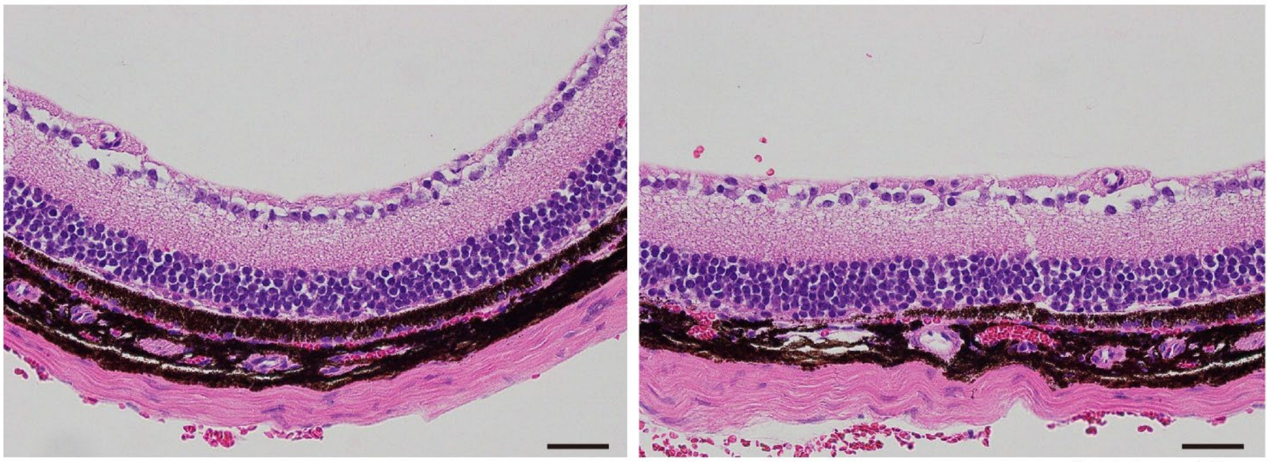

Supplementary Figure 19. H&E staining microscopic image of a 8-weeks-old rd1 mouse retina (Scale bars: 50 μm). This experiment was repeated more than 10 times independently with similar results.

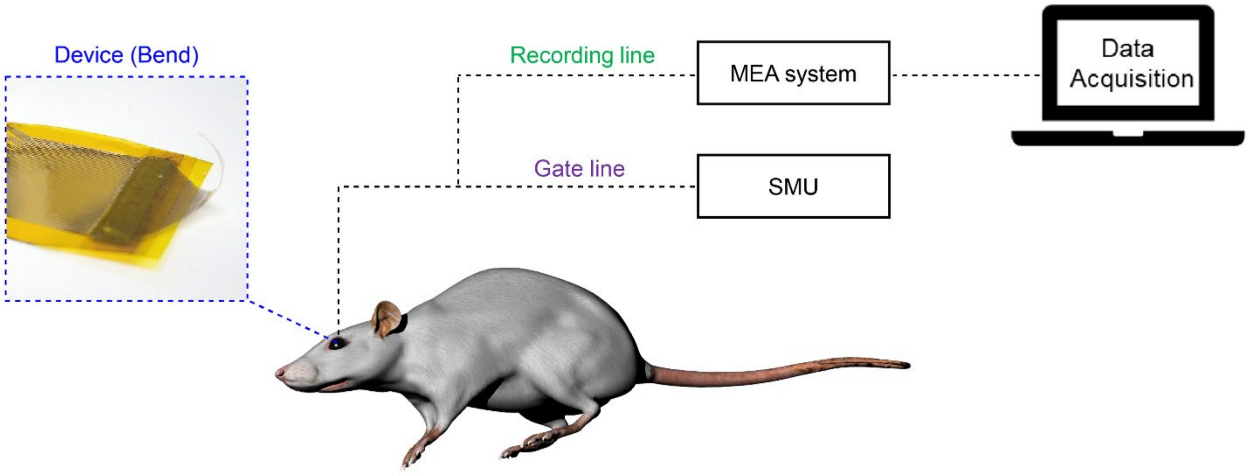

Supplementary Figure 20. Experimental set-up for *in-vivo* animal experiment

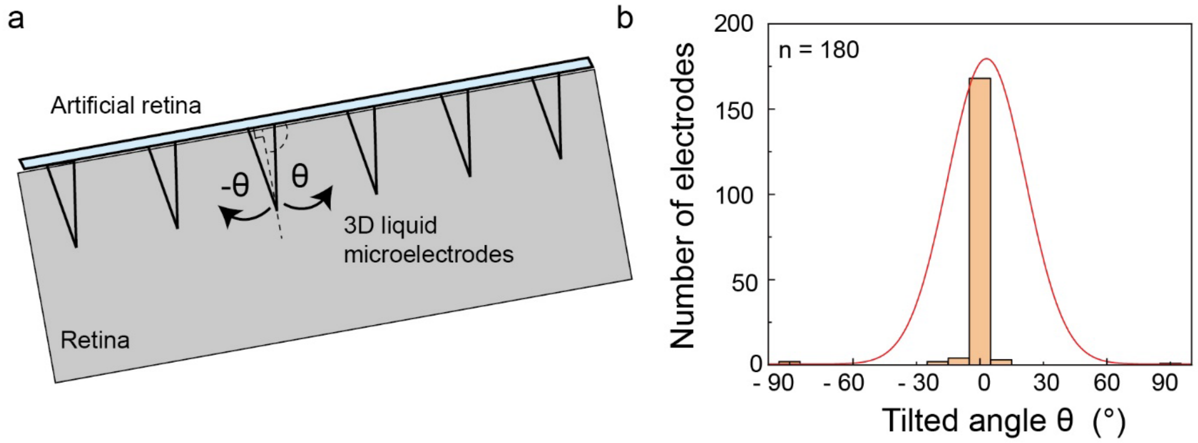

Supplementary Figure 21. a, Schematic illustration defining the tilted angle of the 3D microelectrodes from the OCT image. **b,** Statistical analysis on the tilted angles from the OCT image after implantation (n = 180, 36 pixels per device).

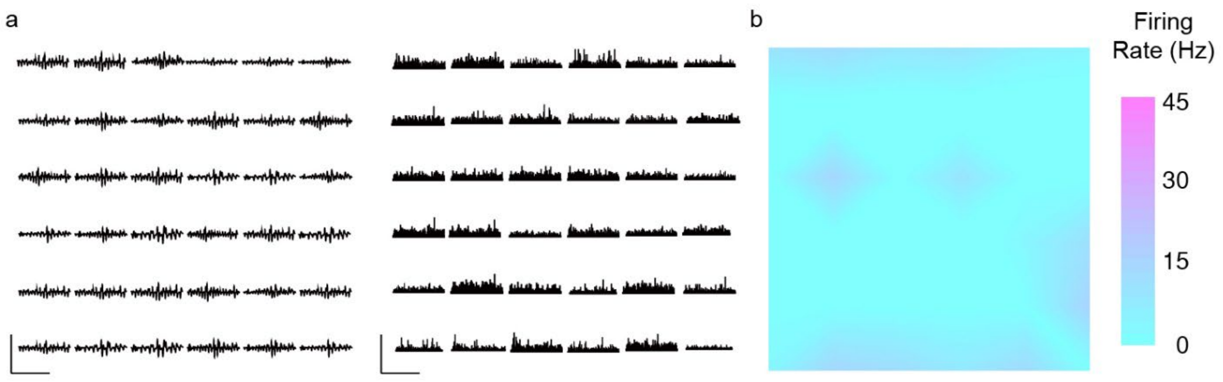

Supplementary Figure 22. a, Retinal responses (spike train and firing rate of evoked RGC spikes) during the operation of our artificial retina device with no light illumination. Scale bars, horizontal 200 ms, vertical 100 μV (Left), horizontal 200 ms, vertical 40 Hz (Right), respectively. **b,** Contour plot of firing rates of evoked RGC spikes with no light illumination.

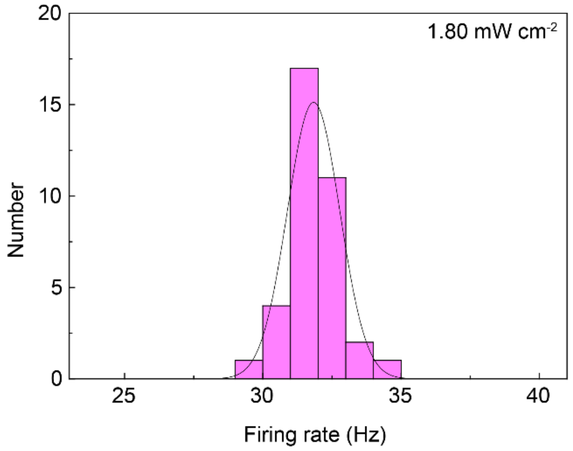

Supplementary Figure 23. Statistical analysis of 36 pixels of the implanted device under the full-field blue light illumination (light intensity of 1.80 mW cm^{-2})

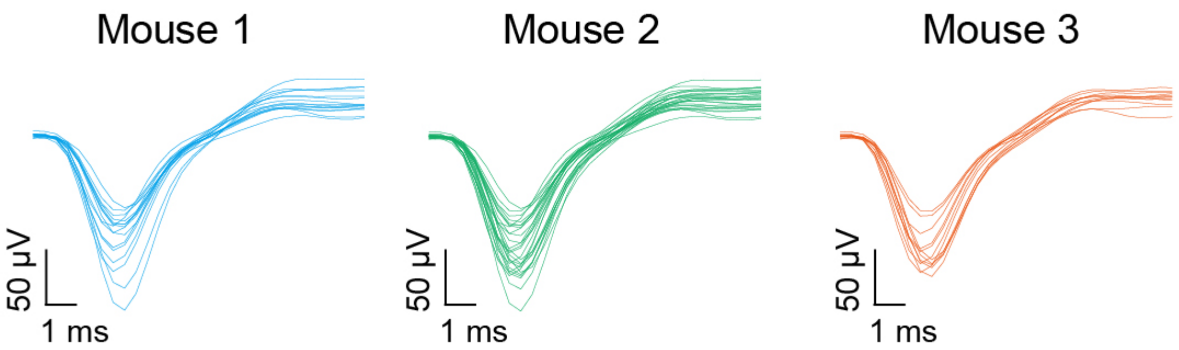

Supplementary Figure 24. Three typical somatic RGC signal-shaped spikes recorded from the local area where the light was illuminated in three rd1 mice.

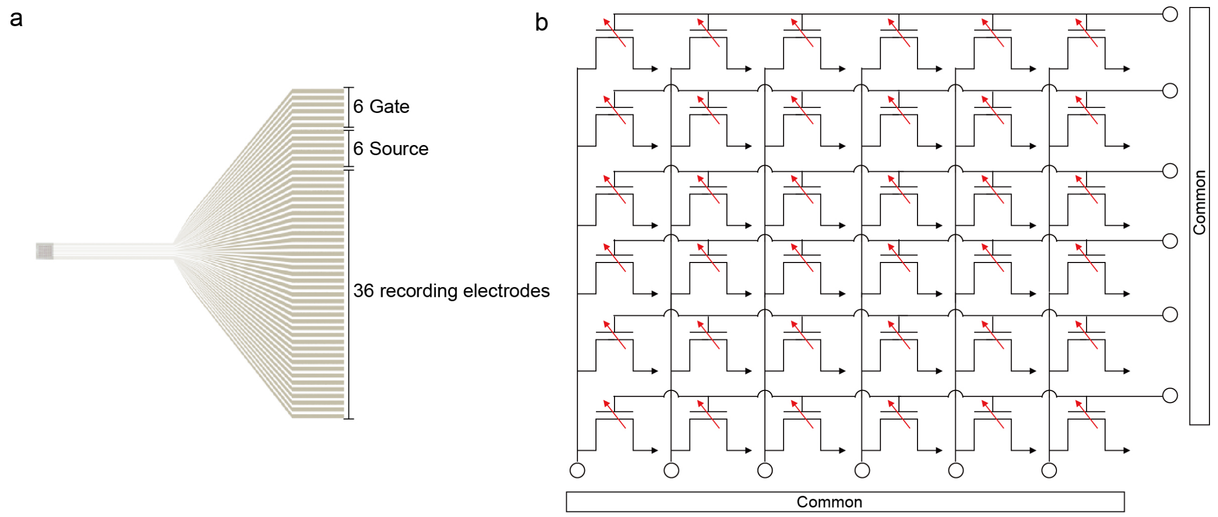

Supplementary Figure 25. a, Design of the device interconnections. **b,** The circuit diagram of the 6 × 6 phototransistor array.

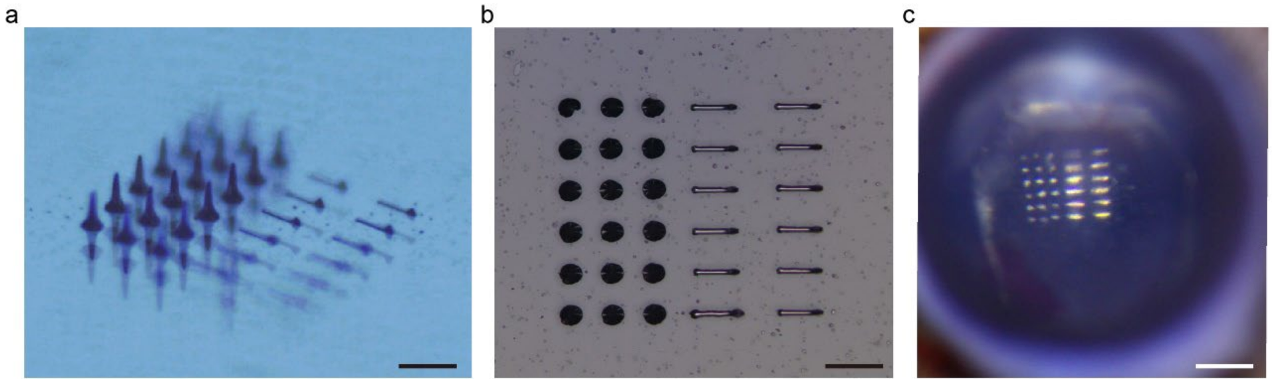

Supplementary Figure 26. a, Photograph of the sample consisting of 18 3D pillars and 12 planar lines of EGaIn. Scale bar, 80 µm. These EGaIn patterns were printed on an 8 mm-thick PI film. **b,** Optical micrograph of this sample. Scale bar, 100 µm. **c,** Fundus image after implanting this sample to the mouse retina. Scale bar, 200 µm.

Layer	Target cells	Thickness	Distance from the inner retinal surface
Retinal nerve fiber layer (NFL)	-	19.12 ± 3.71 μm	0 μm
Ganglion cell/ Inner Plexiform Layer (GCL/IPL)	Ganglion cells	59.62 ± 6.66 μm	19.12 μm
Inner Nuclear Layer (INL)	Bipolar cells	27.82 ± 4.04 μm	78.38 μm
Outer Plexiform Layer (OPL)	-	19.22 ± 4.34 μm	97.6 μm
Outer Nuclear Layer (ONL)	Photoreceptor cells	62.8 ± 6.23 μm	160.4 μm
Retinal Pigment Epithelium (RPE)	-	18.23 ± 2.73 μm	178.63 μm

Supplementary Table 1. Thickness of the retinal layers of the WT mice retina[11].

Supplementary Video legend

Supplementary Video 1. Real-time demonstration of 3D EGaIn pillar using direct printing method.

References

1. Hartigan, J. A. *Clustering Algorithms*. (Wiley, 1975).
2. Nielsen, F. *Introduction to HPC with MPI for Data Science*. (Springer International Publishing, 2016). doi:10.1007/978-3-319-21903-5.
3. Murtagh, F. & Legendre, P. Ward's Hierarchical Agglomerative Clustering Method: Which Algorithms Implement Ward's Criterion? *J. Classif.* **31**, 274–295 (2014).
4. Na, S., Xumin, L. & Yong, G. Research on k-means Clustering Algorithm: An Improved k-means Clustering Algorithm. in *2010 Third International Symposium on Intelligent Information Technology and Security Informatics* 63–67 (2010). doi:10.1109/IITSI.2010.74.
5. Sun, J.-G. Clustering Algorithms Research: Clustering Algorithms Research. *J. Softw.* **19**, 48–61 (2008).
6. Bholowalia, P. & Kumar, A. EBK-Means: A Clustering Technique based on Elbow Method and K-Means in WSN. *Int. J. Comput. Appl.* **105**, 17–24 (2014).
7. Rousseeuw, P. Rousseeuw, P.J.: Silhouettes: A Graphical Aid to the Interpretation and Validation of Cluster Analysis. Comput. Appl. Math. 20, 53-65. *J. Comput. Appl. Math.* **20**, 53–65 (1987).
8. Wang, A., Jung, D., Lee, D. & Wang, H. Impedance Characterization and Modeling of Subcellular to Micro-sized Electrodes with Varying Materials and PEDOT:PSS Coating for Bioelectrical Interfaces. *ACS Appl. Electron. Mater.* **3**, 5226–5239 (2021).
9. Goh, A. et al. Evaluation of Activated Carbon and Platinum Black as High-Capacitance Materials for Platinum Electrodes. *Sensors* **22**, 4278 (2022).
10. Yamagiwa, S. et al. Layer-by-layer assembled nanorough iridium-oxide/platinum-black for low-voltage microscale electrode neurostimulation. *Sens. Actuators B Chem.* **206**, 205-211 (2015).
11. Ferguson, L. R., Dominguez Ii, J. M., Balaiya, S., Grover, S. & Chalam, K. V. Retinal Thickness Normative Data in Wild-Type Mice Using Customized Miniature SD-OCT. *PLoS ONE* **8**, e67265 (2013).
