# Supplementary material for: Liquid-metal-based three-dimensional microelectrode arrays integrated with implantable ultrathin retinal prosthesis for vision restoration
Source: Nat Nanotechnol. 2024 Jan 15;19(5):688–97. doi: 10.1038/s41565-023-01587-w (PMC11106006; doi:10.1038/s41565-023-01587-w)
Supplement: Supplementary file 2 — Reporting Summary [file 41565_2023_1587_MOESM2_ESM.pdf]

Corresponding author(s): Jang-Ung Park
Last updated by author(s): Nov 22, 2023

Reporting Summary

Nature Portfolio wishes to improve the reproducibility of the work that we publish. This form provides structure for consistency and transparency in reporting. For further information on Nature Portfolio policies, see our Editorial Policies and the Editorial Policy Checklist.

Statistics

For all statistical analyses, confirm that the following items are present in the figure legend, table legend, main text, or Methods section.

n/a	Confirmed	
☐	☒	The exact sample size (n) for each experimental group/condition, given as a discrete number and unit of measurement
☐	☒	A statement on whether measurements were taken from distinct samples or whether the same sample was measured repeatedly
☐	☒	The statistical test(s) used AND whether they are one- or two-sided<br>*Only common tests should be described solely by name; describe more complex techniques in the Methods section.*
☒	☐	A description of all covariates tested
☒	☐	A description of any assumptions or corrections, such as tests of normality and adjustment for multiple comparisons
☐	☒	A full description of the statistical parameters including central tendency (e.g. means) or other basic estimates (e.g. regression coefficient) AND variation (e.g. standard deviation) or associated estimates of uncertainty (e.g. confidence intervals)
☐	☒	For null hypothesis testing, the test statistic (e.g. F, t, r) with confidence intervals, effect sizes, degrees of freedom and P value noted<br>*Give P values as exact values whenever suitable.*
☒	☐	For Bayesian analysis, information on the choice of priors and Markov chain Monte Carlo settings
☒	☐	For hierarchical and complex designs, identification of the appropriate level for tests and full reporting of outcomes
☒	☐	Estimates of effect sizes (e.g. Cohen's d, Pearson's r), indicating how they were calculated

Our web collection on statistics for biologists contains articles on many of the points above.

Software and code

Policy information about availability of computer code

Data collection	Electrophysiological recordings of the retina : Multi-electrode array recording and a data processor with a real-time controller (RZ2 BioAmp Processor, Tucker-Davis Technologies, USA)<br>Microsoft Excel 2022
Data analysis	Bandpass filtering and spike detection was made by a custom code with MATLAB R2021a (MathWorks)<br>Origin Pro 2022b (Origin Lab)

For manuscripts utilizing custom algorithms or software that are central to the research but not yet described in published literature, software must be made available to editors and reviewers. We strongly encourage code deposition in a community repository (e.g. GitHub). See the Nature Portfolio guidelines for submitting code & software for further information.

Data

Policy information about availability of data

All manuscripts must include a data availability statement. This statement should provide the following information, where applicable:

- Accession codes, unique identifiers, or web links for publicly available datasets
- A description of any restrictions on data availability
- For clinical datasets or third party data, please ensure that the statement adheres to our policy

Figshare dataset link: https://doi.org/10.6084/m9.figshare.22815461
The custom codes for MATLAB used in this study and the access to our raw data are available from the corresponding authors upon reasonable request.

Human research participants

Policy information about studies involving human research participants and Sex and Gender in Research.

Reporting on sex and gender	*Use the terms sex (biological attribute) and gender (shaped by social and cultural circumstances) carefully in order to avoid confusing both terms. Indicate if findings apply to only one sex or gender; describe whether sex and gender were considered in study design whether sex and/or gender was determined based on self-reporting or assigned and methods used. Provide in the source data disaggregated sex and gender data where this information has been collected, and consent has been obtained for sharing of individual-level data; provide overall numbers in this Reporting Summary. Please state if this information has not been collected. Report sex- and gender-based analyses where performed, justify reasons for lack of sex- and gender-based analysis.*
Population characteristics	*Describe the covariate-relevant population characteristics of the human research participants (e.g. age, genotypic information, past and current diagnosis and treatment categories). If you filled out the behavioural & social sciences study design questions and have nothing to add here, write "See above."*
Recruitment	*Describe how participants were recruited. Outline any potential self-selection bias or other biases that may be present and how these are likely to impact results.*
Ethics oversight	*Identify the organization(s) that approved the study protocol.*

Note that full information on the approval of the study protocol must also be provided in the manuscript.

Field-specific reporting

Please select the one below that is the best fit for your research. If you are not sure, read the appropriate sections before making your selection.

☒ Life sciences ☐ Behavioural & social sciences ☐ Ecological, evolutionary & environmental sciences

For a reference copy of the document with all sections, see nature.com/documents/nr-reporting-summary-flat.pdf

Life sciences study design

All studies must disclose on these points even when the disclosure is negative.

Sample size	The number of animals were chosen enough to perform statistical analysis.
Data exclusions	No data were excluded from the analyses
Replication	Replication of the device was successful.
Randomization	The number of animals were allocated enough to perform statistical analysis as well as the devices.
Blinding	NA

Reporting for specific materials, systems and methods

We require information from authors about some types of materials, experimental systems and methods used in many studies. Here, indicate whether each material, system or method listed is relevant to your study. If you are not sure if a list item applies to your research, read the appropriate section before selecting a response.

Materials & experimental systems

n/a	Involved in the study	
☐	☒	Antibodies
☐	☒	Eukaryotic cell lines
☒	☐	Palaeontology and archaeology
☐	☒	Animals and other organisms
☒	☐	Clinical data
☒	☐	Dual use research of concern

Methods

n/a	Involved in the study	
☒	☐	ChIP-seq
☐	☒	Flow cytometry
☒	☐	MRI-based neuroimaging

Antibodies

Antibodies used

The antibodies used in this study were as follows:
anti-CD68 (Cell Signaling Technology, 97778S, 1:500 dilution)
anti-CD11b (Abcam, ab62817, 1:500 dilution)
anti-SNCG (Abnova, H00006623-M10A, 1:500 dilution)
Donkey anti-Mouse IgG (H+L) Highly Cross-Adsorbed Secondary Antibody, Alexa Fluor™ 594 (Invitrogen, A21203, 1:1000 dilution)
Donkey anti-Rabbit IgG (H+L) Highly Cross-Adsorbed Secondary Antibody, Alexa Fluor™ 488 (Invitrogen, A21206, 1:1000 dilution)
Donkey anti-Goat IgG (H+L) Highly Cross-Adsorbed Secondary Antibody, Alexa Fluor™ Plus 405 (Invitrogen, A48259, 1:1000 dilution)
Rabbit anti-Goat IgG (H+L), Superclonal™ Recombinant Secondary Antibody, Alexa Fluor™ 594 (Invitrogen, A27016, 1:1000 dilution)

Validation

All antibodies used in this study were obtained from the indicated commercial vendors, and the validations of each antibody are provided by the vendor on the products' webpage:
anti-CD68: https://www.cellsignal.com/products/primary-antibodies/cd68-e3o7v-rabbit-mab/97778
anti-CD11b: https://www.abcam.com/products/primary-antibodies/cd11b-antibody-ab62817.html
anti-SNCG: https://www.abnova.com/en-global/product/detail/H00006623-M01A
Donkey anti-Mouse IgG (H+L) Highly Cross-Adsorbed Secondary Antibody, Alexa Fluor™ 594: https://www.thermofisher.com/antibody/product/Donkey-anti-Mouse-IgG-H-L-Highly-Cross-Adsorbed-Secondary-Antibody-Polyclonal/A-21203
Donkey anti-Rabbit IgG (H+L) Highly Cross-Adsorbed Secondary Antibody, Alexa Fluor™ 488: https://www.thermofisher.com/antibody/product/Donkey-anti-Rabbit-IgG-H-L-Highly-Cross-Adsorbed-Secondary-Antibody-Polyclonal/A-21206
Donkey anti-Goat IgG (H+L) Highly Cross-Adsorbed Secondary Antibody, Alexa Fluor™ Plus 405: https://www.thermofisher.com/antibody/product/Donkey-anti-Goat-IgG-H-L-Highly-Cross-Adsorbed-Secondary-Antibody-Polyclonal/A48259
Rabbit anti-Goat IgG (H+L), Superclonal™ Recombinant Secondary Antibody, Alexa Fluor™ 594: https://www.thermofisher.com/antibody/product/Rabbit-anti-Goat-IgG-H-L-Secondary-Antibody-Recombinant-Polyclonal/A27016

Eukaryotic cell lines

Policy information about cell lines and Sex and Gender in Research

Cell line source(s)

ARPE-19 was obtained from ATCC.

Authentication

The authentication of cell lines performed by ATCC can be found at https://www.atcc.org/api/pdf/product-sheet?id=CRL-2302.

Mycoplasma contamination

All cell lines tested negative for mycoplasma.

Commonly misidentified lines
(See ICLAC register)

No commonly misidentified cell lines were used.

Animals and other research organisms

Policy information about studies involving animals; ARRIVE guidelines recommended for reporting animal research, and Sex and Gender in Research

Laboratory animals

Wild-type mice : Male, C57BL/6J, 8-weeks old; Rd1 mice : Male, C3H, 8-weeks old

Wild animals

n/a

Reporting on sex

This study involved only male for both wild-type and rd1 mice to exclude sex-related variations.

Field-collected samples

n/a

Ethics oversight

All of the experimental procedures performed on the animals were conducted based on the guidelines and were approved by the Institute of Animal Care and Use Committee of Yonsei University.

Note that full information on the approval of the study protocol must also be provided in the manuscript.

Flow Cytometry

Plots

Confirm that:

☒ The axis labels state the marker and fluorochrome used (e.g. CD4-FITC).

☒ The axis scales are clearly visible. Include numbers along axes only for bottom left plot of group (a 'group' is an analysis of identical markers).

☒ All plots are contour plots with outliers or pseudocolor plots.

☒ A numerical value for number of cells or percentage (with statistics) is provided.

Methodology

Sample preparation	The artificial retina device and PI film were each cut into 2 mm x 1.5 mm pieces and attached to a 96-well cell culture plate. human retinal pigmented epithelium cells (Human ARPE19 cells) seeded with 3,000 on the reference, negative control (PI film), positive control (puromycin-treated cells), and artificial retina and cultured at 37 °C, 5% CO_2 for 7 days. The positive control was treated with puromycin at a concentration of 0.5 μg/ml. To harvest cultured cells on artificial retinal devices and PI films, the devices and films were detached with forceps, transferred to 1.5 ml tubes, and treated with 0.25% trypsin/EDTA The reference and puromycin-treated positive controls were harvested by treating the cells on the plates with 0.25% T/E. Harvested cells were double stained using the Annexin V conjugated with fluorescein isothiocyanate (FITC) and propidium iodide in the kit (Annexin V-FITC Apoptosis Detection kit, Sigma-Aldrich) for 10 minutes in the dark at room temperature.
Instrument	Flow cytometry analysis was performed by using BD FACS Verse II (Becton Dickinson and company)
Software	Data were acquired in BD FACSuite software and analysed in FlowJo software.
Cell population abundance	For analysis, 10,000 cells were recorded from each sample.
Gating strategy	Cells were first gated to exclude debris (using FSC-A vs SSC-A), then gated for singlet (using FSC-H vs FSC-W and SSC-H vs SSC-W, sequentially). Gating was set up to use reference and positive control (puromycin-treated cells) (PI negative vs PI positive and FITC negative vs FITC positive, respectively).

☒ Tick this box to confirm that a figure exemplifying the gating strategy is provided in the Supplementary Information.

March 2021
