# Supplementary material for: Liquid-metal-based three-dimensional microelectrode arrays integrated with implantable ultrathin retinal prosthesis for vision restoration
Source: Nat Nanotechnol. 2024 Jan 15;19(5):688–97. doi: 10.1038/s41565-023-01587-w (PMC11106006; doi:10.1038/s41565-023-01587-w)
Supplement: Supplementary file 3 — Thickness of the retinal layers of the WT mice retina. [file 41565_2023_1587_MOESM3_ESM.docx]

| **Layer** | **Target cells** | **Thickness** | **Distance from the inner retinal surface** |
| --- | --- | --- | --- |
| Retinal nerve fiber layer (NFL) | - | 19.12 ± 3.71 μm | 0 μm |
| Ganglion cell/ Inner Plexiform Layer (GCL/IPL) | Ganglion cells | 59.62 ± 6.66 μm | 19.12 μm |
| Inner Nuclear Layer (INL) | Bipolar cells | 27.82 ± 4.04 μm | 78.38 μm |
| Outer Plexiform Layer (OPL) | - | 19.22 ± 4.34 μm | 97.6 μm |
| Outer Nuclear Layer (ONL) | Photoreceptor cells | 62.8 ± 6.23 μm | 160.4 μm |
| Retinal Pigment Epithelium (RPE) | - | 18.23 ± 2.73 μm | 178.63 μm |

**Supplementary Table 1.** Thickness of the retinal layers of the WT mice retina^1^.

**References**

1. Ferguson, L. R., Dominguez Ii, J. M., Balaiya, S., Grover, S. & Chalam, K. V. Retinal Thickness Normative Data in Wild-Type Mice Using Customized Miniature SD-OCT. *PLoS ONE* **8**, e67265 (2013).
