# Supplementary figures and images for: Liquid-metal-based three-dimensional microelectrode arrays integrated with implantable ultrathin retinal prosthesis for vision restoration
Source: Nat Nanotechnol. 2024 Jan 15;19(5):688–97. doi: 10.1038/s41565-023-01587-w (PMC11106006; doi:10.1038/s41565-023-01587-w)

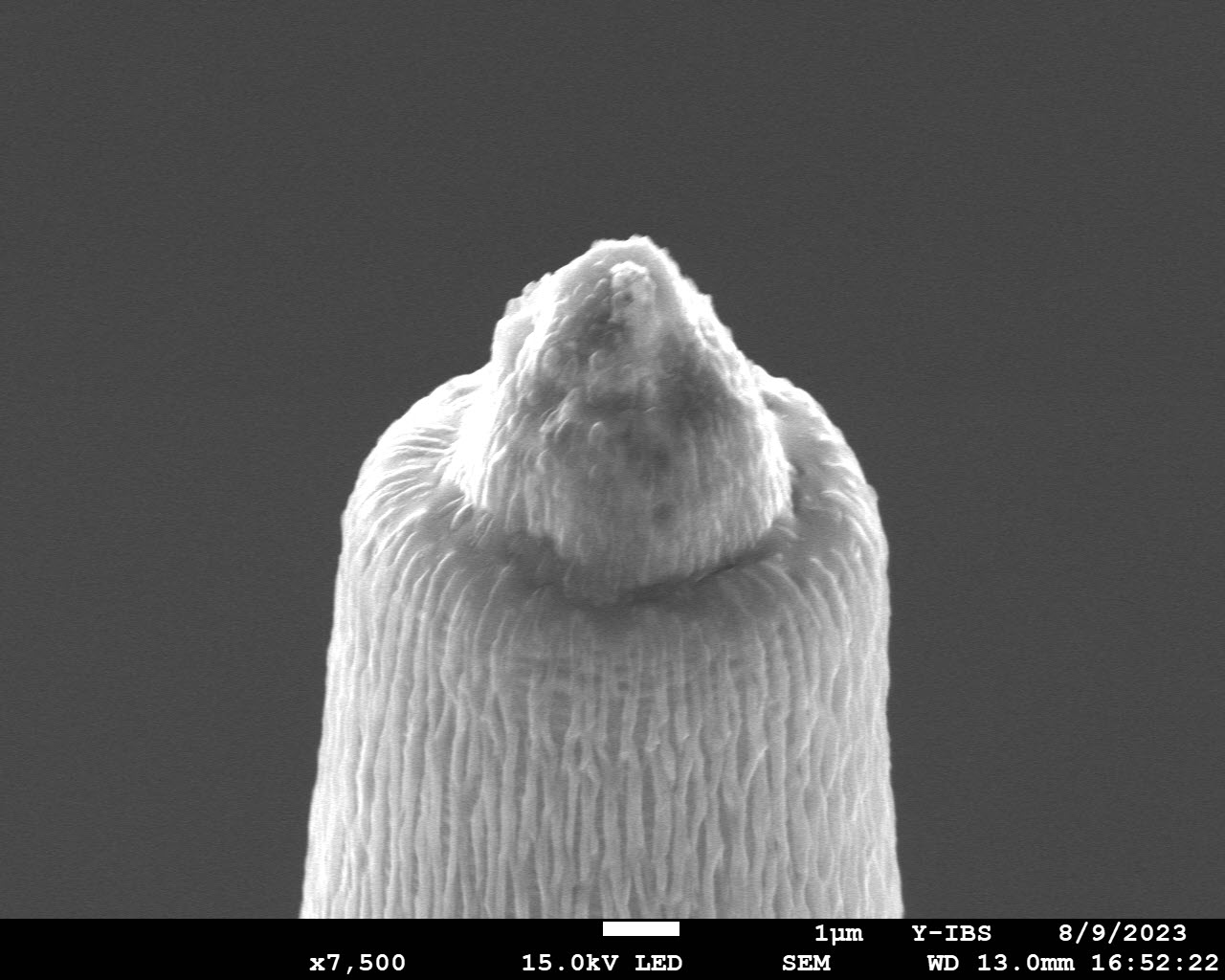

Supplement: Supplementary file 9 — SEM images. [file 41565_2023_1587_MOESM9_ESM.zip › Extended Data Fig. 1/x7500.jpg]

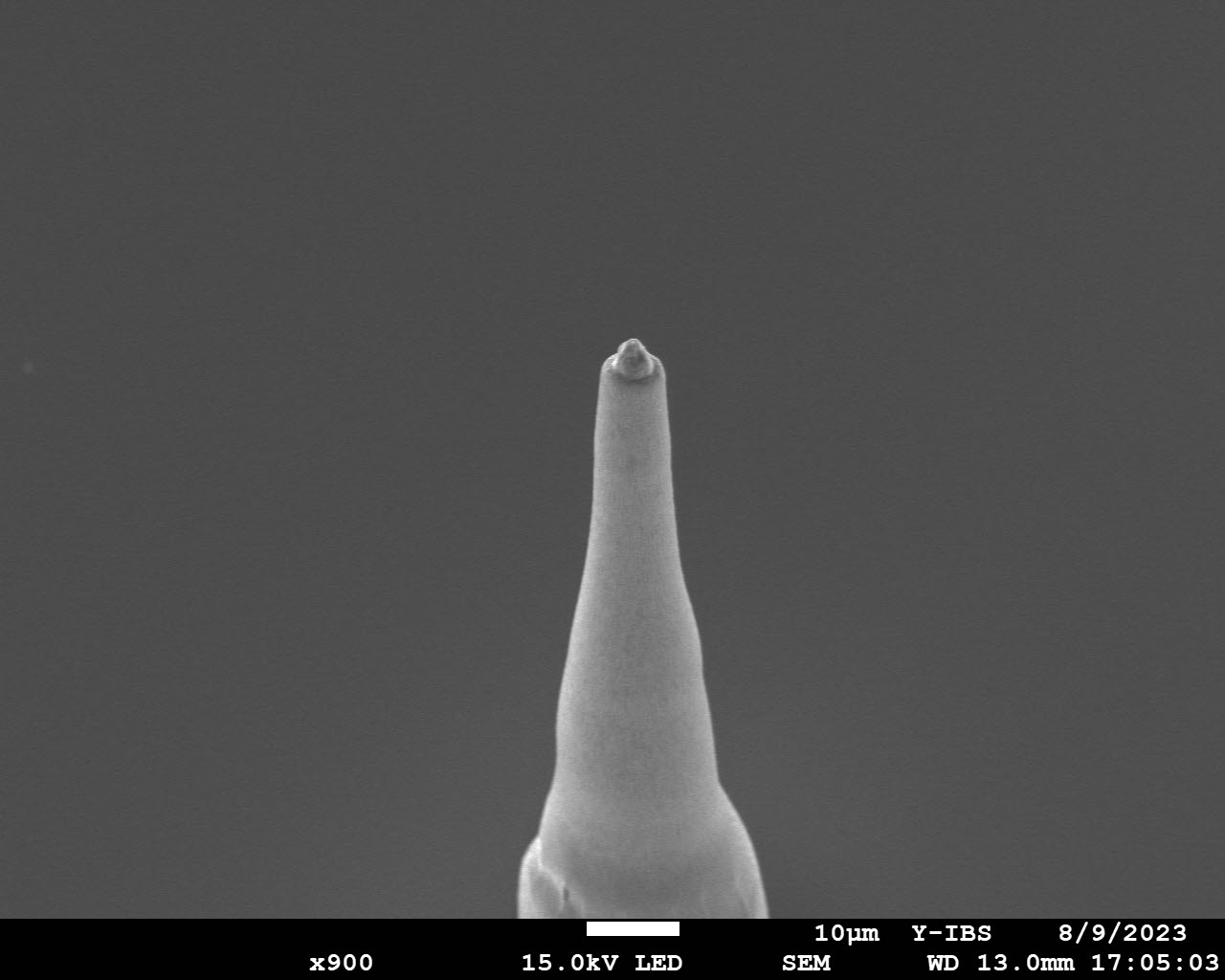

Supplement: Supplementary file 9 — SEM images. [file 41565_2023_1587_MOESM9_ESM.zip › Extended Data Fig. 1/x900.jpg]

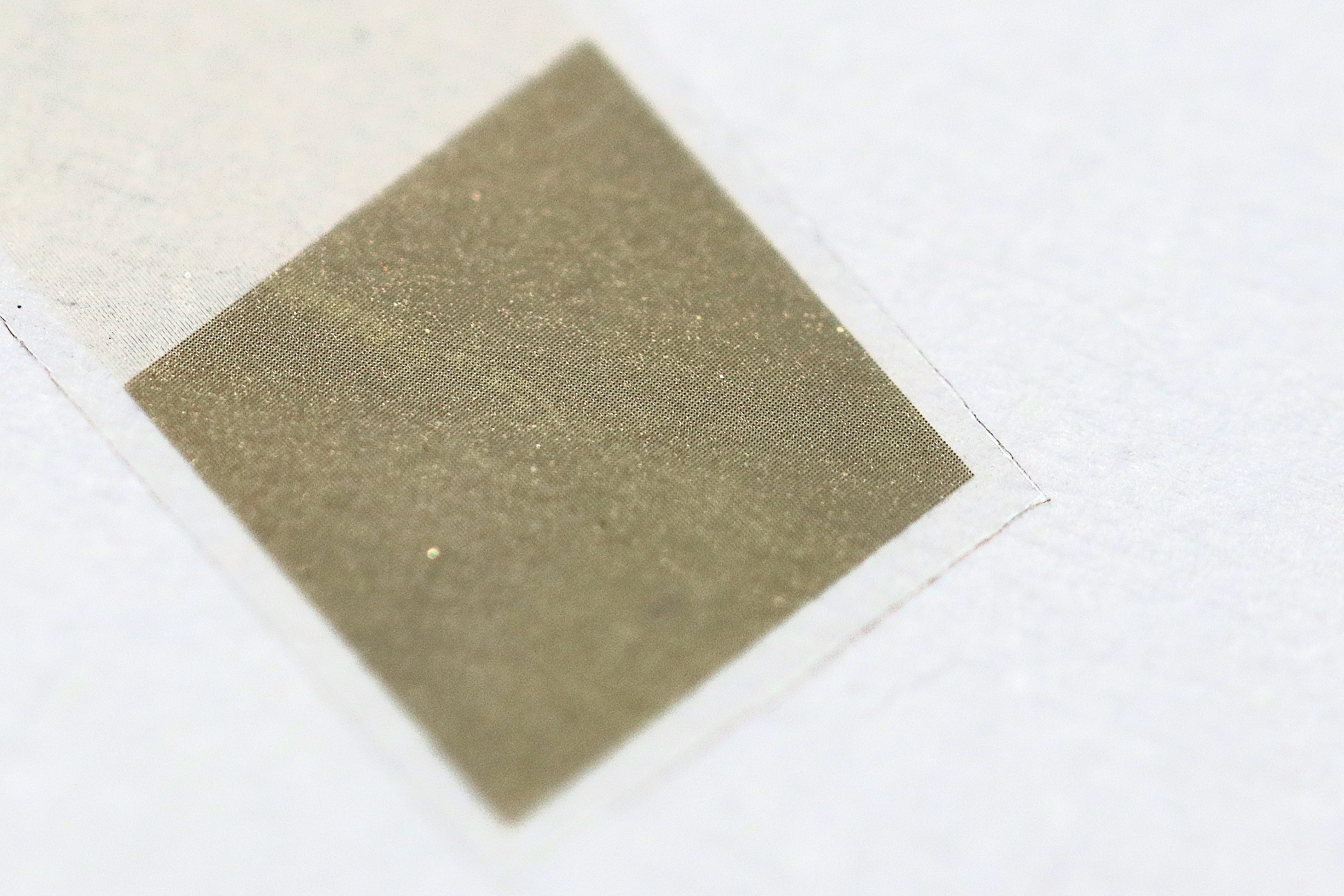

Supplement: Supplementary file 10 — Photograph and SEM images. [file 41565_2023_1587_MOESM10_ESM.zip › Extended Data Fig. 5/Device image.jpg]

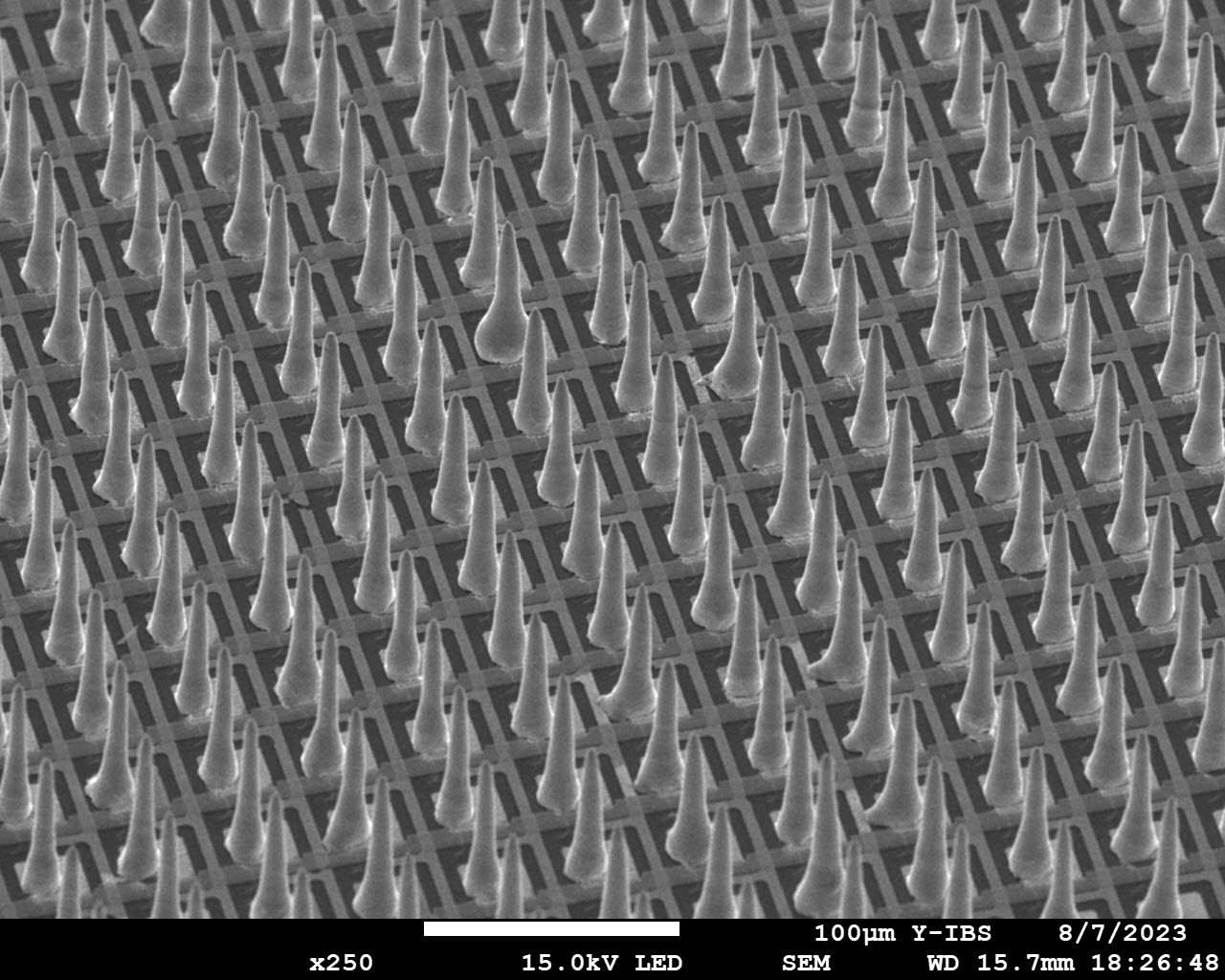

Supplement: Supplementary file 10 — Photograph and SEM images. [file 41565_2023_1587_MOESM10_ESM.zip › Extended Data Fig. 5/SEM image.jpg]
